# Characterization and virulence of yeasts associated with neonatal thrush

**DOI:** 10.1186/s12866-025-04075-4

**Published:** 2025-06-26

**Authors:** Eman G. A. M. El-Dawy, Abeer Baddar, Youssuf A. Gherbawy, Asmaa S. Yassein

**Affiliations:** 1 Botany and Microbiology Department, Faculty of Science, South Valley University, Qena, 83523 Egypt; 2 Applied and Environmental Microbiology Center, South Valley University, Qena, Egypt

**Keywords:** Neonatal thrush, *Candida* species, *PLB1* gene, *SAP1*gene

## Abstract

**Background:**

Species of *Candida* are the main cause of aggressive fungal infections in preterm newborns. These infections are linked to significant illness and death. Neonatal thrush is diagnosed by observing white patches on the surfaces of the mucosa of the mouth. Yeast is often associated with it. This study aimed to apply different techniques to characterize the yeasts associated with neonatal mouth thrush. Laboratory-based yeast species identification techniques (morphological and molecular) have been employed. Virulence genes were tested to compare experimental virulence assays and the presence of studied genes.

**Results:**

Our results indicated that forty-one isolates out of 79 were identified as *C. albicans* and confirmed by using (*CALB1-CALB2*) specific primers. *C. dubliniensis*, *C. glabrata* (*Nakaseomyces glabratus*), *C. guilliermondii* (*Meyerozyma guilliermondii*), *C. krusei* (*Pichia kudriavzevii*), *C. lusitaniae* (*Clavispora lusitaniae*), *C. parapsilosis*, *C. tropicalis*, *C. kefyr* (*Kluyveromyces marxianus*), and *Trichosporon asahii* were also verified by applying (*CandF - CandR*) primer and Internal transcribed spacer (ITS) sequencing. Thirty-six isolates were active producers of the three tested enzymes: hemolysin, phospholipase, and proteinase. Fifty-five isolates secreted hemolysin, and sixty-four isolates were positive for the presence of the phospholipase B1 (*PLB1*) gene. The secreted aspartyl protease 1 (*SAP1*) gene was detected in thirty-three isolates, from which thirty-two isolates secreted the proteinase enzyme.

**Conclusion:**

Different techniques were required to diagnose the *Candida* species and other yeasts associated with neonatal thrush. *C. albicans* was the dominant species. The identification of two species of *C. albicans* and *C. dubliniensis* by specific primer (*CALB1-CALB2*), general primer (*CandF - CandR*), and sequencing techniques was applied in this study.

## Background

The human oral cavity is a distinct location inhabited by over 700 different microbial species, including mycoplasma, viruses, bacteria, fungi, and protozoa. These microorganisms are acquired within the first six hours after birth [1]. Species of *Candida* are opportunistic pathogens that reside in the oral cavity of healthy people, particularly in babies, as commensal, nonthreatening microorganisms under normal circumstances [2]. Dysbiosis, an imbalance in the kinds and distribution of the human body’s natural microflora, is closely linked to various human disorders [3]. Candidiasis of the mouth is known as thrush, which appears on top of the tongue, the inner side of the mouth, and surrounding the lips as white patches. Below these patches, there is an inflamed surface. Also, the edge of the lips can be prone to rupture, and showing a bleeding area might cause slight pain [4].

According to reports, oral thrush affects approximately 37% of newborns under the age of six months and 54% of elderly individuals who wear dentures. Also, postpartum women and pregnant women are more susceptible to oral candidiasis than non-pregnant women due to hormonal changes [5–7].

Neonatal thrush, also known as oral candidiasis, oropharyngeal candidiasis, pseudomembranous candidiasis, or Monilia, is a superficial infection that affects the oral cavity of infants. It manifests as white spots on the tongue and oral mucosa, with red tissue underneath that is susceptible to easy bleeding [8–10]. This occurs due to their undeveloped immune system, which exposes them to acute infections [11]. Transmission of *Candida* spp. from mother to her baby during parturition is called vertical transmission. In contrast, transmission through external factors (ex, contaminated tools) is called horizontal transmission [12, 13].

Most clinical infection isolates, particularly over 80%, consist of *C. albicans*,* C. glabrata*, and *C. tropicalis.* Additionally, other species such as *C. pseudotropicalis*,* C. guillermondii*,* C. krusei*,* C. lusitaniae*,* C. parapsilosis*, and *C. stellatoidea* have been recovered from the oral cavity of newborns [14, 15]. *C. albicans* is the most hazardous species in hospitalized patients and is responsible for invasive candidiasis with a mortality rate of about 50% [16, 17]. The pathogenesis of *Trichosporon asahii* isolated from intensive care unit (ICU)-admitted patients is like that commonly exhibited during the infection of *Candida*. It can be isolated from the oral cavity and urine of patients residing in the ICU over six months [18].

The pathogenicity of *Candida* spp. is interrelated to appointed virulent factors such as biofilm formation, adhesion, ability to avoid host defense, and the secretion of extracellular enzymes like hemolysin, phospholipases, and proteases [19]. These virulence factors are necessary to understand the complications of oral candidiasis to develop proper treatment [20].

*Candida albicans* can produce a little, thin, tube-like structure that is called a germ tube, and it’s supposed to be the most important characteristic of clinical identification and pathogenesis of *C. albicans* when inoculated into human serum and then incubated at 35–37 °C for 2–4 h [21]. The germ tube represents the early step of hyphal initial formation after the elongation of yeast cells [22]. *C. dubliniensis* is like *C. albicans* in morphology and the ability to form germ tubes [23].

The most important virulence factor is hemolytic activity; hemolysin enzyme enables *Candida* to obtain iron from host tissue and use it for its metabolism, growth, and invasion [24]. Iron is found in the oral cavity in different forms, extracellular iron is closely linked to lactoferrin, a protein that exists in saliva, whereas intracellular protein is stored in the form of ferritin, despite that the risk of infections with *C. albicans is* repeated and that assumes the capability of taking iron from the oral cavity in different forms by this yeast [25].

Phospholipase enzymes facilitate the breakdown of the primary component of the cell membrane by hydrolysis in humans (phospholipids) and one or more ester linkages of glycerophospholipids by the cleavage of specific and different ester bonds, phospholipase enzymes have been identified into seven genes (*PLA*, *PLB1*, *PLB2*, *PLC1*, *PLC2*, *PLC3*, and *PLD1*), classified into four categories (A, B, C & D) and only four of them have been well recognized (*PLB1*, *PLB2*, *PLC1*, and *PLD1*), despite that, extracellularly, only the products of PLB1 & PLB2 genes have been identified, *PLB* has fatty acid release (hydrolase) and lysophospholipase transacylase activities together that makes it the principle activity of phospholipases in *Candida* spp. in hyphal form the activity is the most by direct connection [26–30]. It was believed that enzymes secretion by *Candida* spp. such as phospholipase and proteinase, promote the overgrowth of *Candida* as they have a significant role in facilitating their adhesion and penetration into host cells [31].

*Candida* spp., particularly *C. albicans*, secrete aspartic proteases (*Saps*) to consume nutrients and persist in host cells as a natural adaptation of pathogens [32]. *Saps* can destroy numerous human proteins at the site of infection, including extracellular matrix, host surface proteins, and various host defense proteins [33].

This study aimed to find the phenotypic and genotypic characterization of *Candida* species and other yeast-like structures causing oral thrush in neonates, focusing on the correlation between virulent factors.

## Materials and methods

### Reagents

Sabouraud dextrose agar medium (40 g dextrose (OXFORD, 50-99-7); 10 g peptone (ALPHA CHEMIKA, 73049-73-7); 15 g agar, 0.5 g cycloxamide/L of distilled water.

Potato dextrose agar (200 g potatoes; 20 g dextrose, 20 g agar, 250 mg chloramphenicol/L of distilled water).

Chromogenic agar medium (CONDA, Madrid, Spain, Cat.138200).

HiChrome™ *Candida* Differential Agar (Himedia M1297, India).

PSB (200 g/600 mL distilled water; 20 g of sucrose). (*CALB1-CALB2*) primer, (*CandF - CandR*) primer, (*PLB1*, and *SAP1* genes) primers and 2X Crystal Taq Master Mix (Jena Bioscience). Agarose gel in 1x TAE buffer using ethidium bromide (Sigma Aldrich).

CTAB (Sigma Aldrich).

The egg yolk agar plate medium was prepared by adding 5.5 g CaCl_2_ and 58.4 g NaCl to 950 mL of Sabouraud sucrose agar.

Casein hydrolysis medium was prepared by adding 1.0 g KH_2_PO_4_, 0.5 g KCl, 0.2 g MgSO_4_.7H_2_O, 0.1 g CaCl_2_.2H_2_O (L.F.C),10 g sucrose, and 15 g Agar to 1 L of distilled water.

## Methods

### Sample collection

One hundred and seventy-two samples, comprising 93 samples from males and 79 from females, were collected from the oral cavity (tongue and palate) of infected neonates admitted to various intensive care units in Qena Governorate, aged between 2 days and 28 days, during the period from June 2022 to December 2022 (Table 1). Sterilized swabs were used, which were immediately covered and sent directly to the mycology laboratory at the Faculty of Science for testing.

**Table 1:** Detailing the isolated samples showing the gender, the age, and the reasons for hospitalization in ICU

Code	Gender	Age (days)	Reason for hospitalization in ICU
AEMCA1	Female	6	Jaundice
AEMCA2	Male	6	Prematurity
AEMCA3	Male	28	Prematurity
AEMCA4	Female	4	Prematurity
AEMCA5	Male	22	Extreme Low Weight
AEMCA6	Male	2	Jaundice & Prematurity
AEMCA7	Male	3	Prematurity
AEMCA8	Female	1	Prematurity
AEMCA9	Female	9	Prematurity
AEMCA10	Female	2	Jaundice
AEMCA11	Female	8	Extreme Low Weight
AEMCA12	Male	3	Prematurity
AEMCA13	Male	4	Prematurity
AEMCA14	Male	3	Prematurity
AEMCA15	Male	3	Prematurity
AEMCA16	Female	6	Prematurity
AEMCA17	Female	4	Jaundice
AEMCA18	Male	5	Jaundice
AEMCA19	Female	5	Prematurity
AEMCA20	Male	8	Prematurity
AEMCA21	Male	26	irregular heart rhythms & Jaundice
AEMCA22	Male	17	Jaundice
AEMCA23	Male	21	Infection
AEMCA24	Male	4	Jaundice
AEMCA25	Male	3	Extreme Low Weight
AEMCA26	Female	25	Jaundice & Prematurity
AEMCA27	Female	6	breech delivery
AEMCA28	Female	6	Prematurity
AEMCA29	Male	4	Prematurity
AEMCA30	Male	9	Prematurity
AEMCA31	Male	13	respiratory distress syndrome
AEMCA32	Female	27	Extreme Low Weight
AEMCA33	Female	8	Extreme Low Weight
AEMCA34	Female	5	Extreme Low Weight
AEMCA35	Male	15	Extreme Low Weight
AEMCA36	Male	7	Extreme Low Weight
AEMCA37	Female	28	Anemia
AEMCA38	Male	3	Jaundice
AEMCA39	Female	1	Jaundice & Extreme Low Weight
AEMCA40	Male	22	Prematurity
AEMCA41	Male	28	Prematurity
AEMCD42	Male	6	Extreme low Weight
AEMCD43	Female	6	Infection
AEMCD44	Female	6	Extreme Low Weight
AEMCD45	Male	6	Infection
AEMCD46	Female	6	jaundice
AEMCD47	Female	28	Extreme Low Weight
AEMCD48	Male	15	Jaundice
AEMCD49	Male	2	Jaundice
AEMCD50	Female	15	Jaundice
AEMCD51	Male	9	Extreme Low Weight
AEMCD52	Male	8	Infection
AEMCD53	Male	7	Resuscitation at Birth
AEMCGL54	Female	2	Jaundice
AEMCGL55	Female	6	Prematurity
AEMCGL 56	Female	15	Extreme Low Weight
AEMCGL57	Male	9	Extreme Low Weight
AEMCGU58	Male	4	Extreme Low Weight & infection
AEMCKR59	Male	5	Extreme Low Weight
AEMCKR60	Female	6	Jaundice & Prematurity
AEMCKR61	Male	6	Extreme Low Weight
AEMCKR62	Male	3	Prematurity
AEMCKR63	Male	5	Infection
AEMCKR64	Female	5	Respiratory distress syndrome
AEMCKR65	Female	9	Prematurity
AEMCKR66	Female	10	Jaundice & infection
AEMCKR67	Male	3	Extreme Low Weight
AEMCKR68	Male	6	Jaundice
AEMCKR69	Male	10	breech delivery
AEMCL70	Male	2	Jaundice
AEMCP71	Female	6	Prematurity
AEMCT72	Female	5	Extreme Low Weight
AEMCT73	Female	12	Respiratory distress syndrome
AEMCT74	Male	4	Jaundice
AEMCT75	Female	3	Jaundice & Prematurity
AEMCT76	Male	1	Prematurity
AEMCT77	Male	8	Extreme Low Weight
AEMKM78	Female	8	Jaundice & Prematurity
AEMTA79	Male	5	Extreme Low Weight

### Culturing samples

The dilution technique was used for culturing the specimens on Sabouraud dextrose agar medium [34, 35], then the cultures were incubated for 48 h at 37 °C. After the appearance of colonies, they were purified and subcultured on Potato Dextrose Agar (PDA) as described by Gams et al. [36] with little modification slants and kept in the fridge for further processing.

### Morphological identification of the isolated yeasts

*Candida* Chromogenic agar medium is a differential and selective medium, obtained from CONDA Company (Madrid, Spain, Cat.138200). It was utilized for the rapid identification of *Candida* spp. after incubation for 48 h. Each of the four species of yeast, namely *C. albicans*, *C. glabrata* (*Nakaseomyces glabratus*), *C. krusei* (*Pichia kudriavzevii*), and *C. tropicalis*, displayed distinct variations in colony color on the medium [37, 38]. HiChrome™ *Candida* Differential Agar (Himedia M1297, India) is a selective medium used for direct identification of four species of yeast (*C. albicans*, *C. glabrata* (*Nakaseomyces glabratus*), *C. krusei* (*Pichia kudriavzevii*) & *C. tropicalis*) throughout 48 h. *C. albicans* could produce β-N-acetyl-galactosaminidase enzyme, as mentioned by Perry and Miller [39], the incorporation of the fluorogenic hexosaminidase substrates into the medium is the basis of *C. albicans* identification [40]. The germ tube test was carried out as described by Makwana et al. [41]. Human serum samples intended for disposal in the safety box in Al-Quds lab at Naqda, Qena, Egypt, for medical analysis were collected with the explicit approval of the laboratory owner. These were utilized following ethical guidelines and solely for research purposes. The serum samples were collected during the period from April 2023 to June 2023. A 0.5 ml portion of the serum was inoculated with 2–3 *Candida* colonies and incubated at 37 °C for 2–3 h.

The morphological aspects were analyzed by Principal Component Analysis (PCA), OriginPro 2019b 64-bit.

### Molecular identification

#### Extraction of DNA

For DNA extraction, the strains were cultured in 5 mL of Potato Sucrose Broth (PSB) (200 g/600 mL distilled water; 20 g of sucrose) and incubated at 37 ◦C for 48–72 h. The yeasts were isolated from the medium using centrifugation at a speed of 3000 rpm for 5 min. The liquid portion above the yeast cells was discarded, and the Cetyltrimethylammonium bromide (CTAB) technique, as described by Doyle and Doyle [42], was employed.

A specific primer was used for the identification of *C. albicans* that was designed according to White et al. [43]. Two oligonucleotides (*CALB1-CALB2*) (Jena Bioscience) were created using the sequence data for the internal transcribed spacer (ITS) region. The forward and reverse oligonucleotides (*CALB1 and CALB2*) (Jena Bioscience) were designed as a part of the ITS1 and ITS2 regions (Table 2). Another pair of primers (*CandF - CandR*) (Jena Bioscience) was designed based on the analysis of a specific region (18 S-ITS1-5.8 S-ITS2-28 S) in the rDNA of *Candida* spp. that placed in the GenBank to identify eight species of *Candida* (*C. albicans*, *C. dubliniensis*, *C. glabrata/Nakaseomyces glabratus*, *C. guilliermondii/Meyerozyma guilliermondii*, *C. krusei*/*Pichia kudriavzevii*, *C. lusitaniae*/*Clavispora lusitaniae*, *C. parapsilosis*, and *C. tropicalis*) using simplex PCR based on the amplicon size, showing a detection limit of 10 pg/µL of DNA or 10^3^ yeasts/mL [44]. The sequencing of the ITS region was employed for some isolates to confirm the identification. The reaction was done in 20 µl as a finalized volume that consisted of 10 µl of 2X Crystal Taq Master Mix (Jena Bioscience), 1.0 µl DNA template, 0.5 µl of each specific primer, and distilled water was added to complete the volume. The amplification program was carried out as it was designed by Zhang et al. [45]. The electrophoresis was applied according to Moeller et al. [46] on a 1.4% agarose gel in 1x TAE buffer using ethidium bromide (Sigma Aldrich) as a stain to separate the PCR products, and 100 bp DNA ladder (Jena Bioscience) was used as a marker. Finally, the Gel Doc UVP Bioimaging system (GPS-8000 system) was applied for scanning. The resulting sequences were deposited in GenBank with accession numbers listed in Fig. 6. A phylogenetic tree of the ITS region sequences was constructed using the MEGA6 software package24. Maximum likelihood analyses were applied using the Tamura–Nei model25. To detect the support for each clade, a bootstrap analysis was performed with 1000 replications.

**Table 2:** Oligonucleotides used for the identification of eight species of *Candida* And the detection of phospholipase B1 And aspartyl proteinase genes in *Candida* spp. And some yeast like–structures

Primer name	Sequence	Species identified by primer	Amplicon size (bp)
*CALB1* *CALB2*	5’-TTT ATC AAC TTG TCA CAC CAG A-3’ 5’-ATC CCG CCT TAC CAC TAC CG-3’	*Candida albicans*	273
*CandF* *CandR*	5’-AGCTTGCGTTGATTACGTCCCTGCCC-3’ 5’-TTCACTCGCCGCTACTAAGGCAATCCC-3’	*C. albicans* *C.dubliniensis* *C. glabrata* (*N. glabratus*) *C. guillermondii* (*M. guilliermondii*) *C. krusei* (*P. kudriavzevii*) *C.lusitaniae* (*C.lusitaniae*) *C. parapsilosis* *C. tropicalis*	850 810 1000 1100 800 590 731 790
*PLB1*	5’-CCT ATT GCC AAA CAA GCA TTG TC-3’ 5’-CCA AGC TAC TGA TTT CAC CTG CTC C-3		179
*SAP1*	5’-TCA ATC AAT TTA CTC TTC CAT TTC TAA CA-3’ 5’-CCA GTA GCA TTA ACA GGA GTT TTA ATG ACA-3		161

### Enzymatic activities

#### Hemolysin

Hemolysin activity was determined according to Malcok et al. [47] with slight changes. Sabouraud sucrose agar medium was used by the addition of 7% defibrinated human blood after cooling to 45–50 °C and supplemented with 3% sucrose, then about an 8 mm disc of growing yeasts was spot inoculated into the medium and incubated at 37 °C for 48 h. A halo of lysis appeared around the colony and was measured by a ruler. The assay was performed in triplicate for each isolate in three separate plates.

#### Phospholipase

Phospholipase activity was estimated as described by Price et al. [48] with little modification. Egg yolk agar plate medium can be prepared by adding 5.5 g CaCl_2_ and 58.4 g NaCl to 950 mL of Sabouraud sucrose agar and sterilized at 121 °C in the autoclave for 20 min, then cooled to 45–50 °C. Egg yolk 50% suspension was prepared, then 50 mL of suspension was added to the cooled medium. A disk of 8 mm diameter of yeasts was inoculated on the plate medium and incubated at 37 °C for 5 days. The diameter of the opaque zone precipitated around colonies was determined. Isolates were verified in triplicate using three separate plates.

#### Protease

Proteolytic activity was measured by using casein hydrolysis medium as mentioned by Paterson and Bridge [49] and Ozturkoglu et al. [50] with slight modification. This medium was prepared by adding 1.0 g KH_2_PO_4_, 0.5 g KCl, 0.2 g MgSO_4_.7H_2_O, 0.1 g CaCl_2_.2H_2_O,10 g sucrose, and 15 g Agar to 1 L of distilled water then sterilized in autoclave at 121 °C for 20 min, cooled to 45–50 °C, finally, 25.0 ml of 15% sterilized skimmed milk was added to the cooled medium and poured to sterilized petri dishes. About an 8 mm disk of purified yeast strain was inoculated on the prepared medium and incubated at 37 °C for seven days. The experiment was performed in triplicate. The clear zone around yeast colonies was estimated by using a ruler.

Enzymatic activity (pz) was calculated by using this equation:

$$\mathrm{Pz}=\frac{\text { Colony diameter Zone of precipitation }}{\text { Colony diameter }}$$

Enzymatic activity was categorized into 4 types: Pz value = 0 means that the test was negative, Pz value 1–1.50 mm means low enzyme secretion, 1.51–2.50 mm for moderate enzyme secretion, and 2.51–3.5 mm for high secretion. Negative control for both enzymes was displayed by inoculating the medium with a disk of the same sterilized agar medium. Positive control represented by the positive images in the studies reported by Price et al. [48], Paterson and Bridge [49], and Ozturkoglu et al. [50]. The data were analyzed by SPSS 16 (One Way ANOVA).

#### Genotypic detection of phospholipase B1 (*PLB1*) and secreted aspartyl proteinase (*SAP1*)

*PLB1* and *SAP1* genes were designed according to Naglik et al. [28], and the previously extracted DNA was used to complete two PCR amplifications. The conditions of *PLB1* and *SAP1* genes were initial denaturation at 94ºC for 3 min; followed by 30 cycles of denaturation at 94ºC for 30 s, annealing at 53, and 46 ºC for 30 s, and extension at 72ºC for 30 s; followed by a final elongation step at 72ºC for 10 min. Positive control represented by the size of *PLB1* and *SAP1* gene bands at 179 bp and 161 bp, respectively, as described in the study of Naglik et al. [28], negative control was displayed by adding sterile PCR water.

## Results

Seventy-nine isolates of *Candida* spp. and yeast-like structures were obtained from the collected 172 neonatal thrush samples on Sabouraud dextrose agar medium at 37 ^o^C. These included 45 isolates from male samples and 34 from females. The collected isolates were subjected to morphological and molecular characterization for accurate identification.

### Distinctions in the morphological features among the isolated samples

**Fig. 1:**
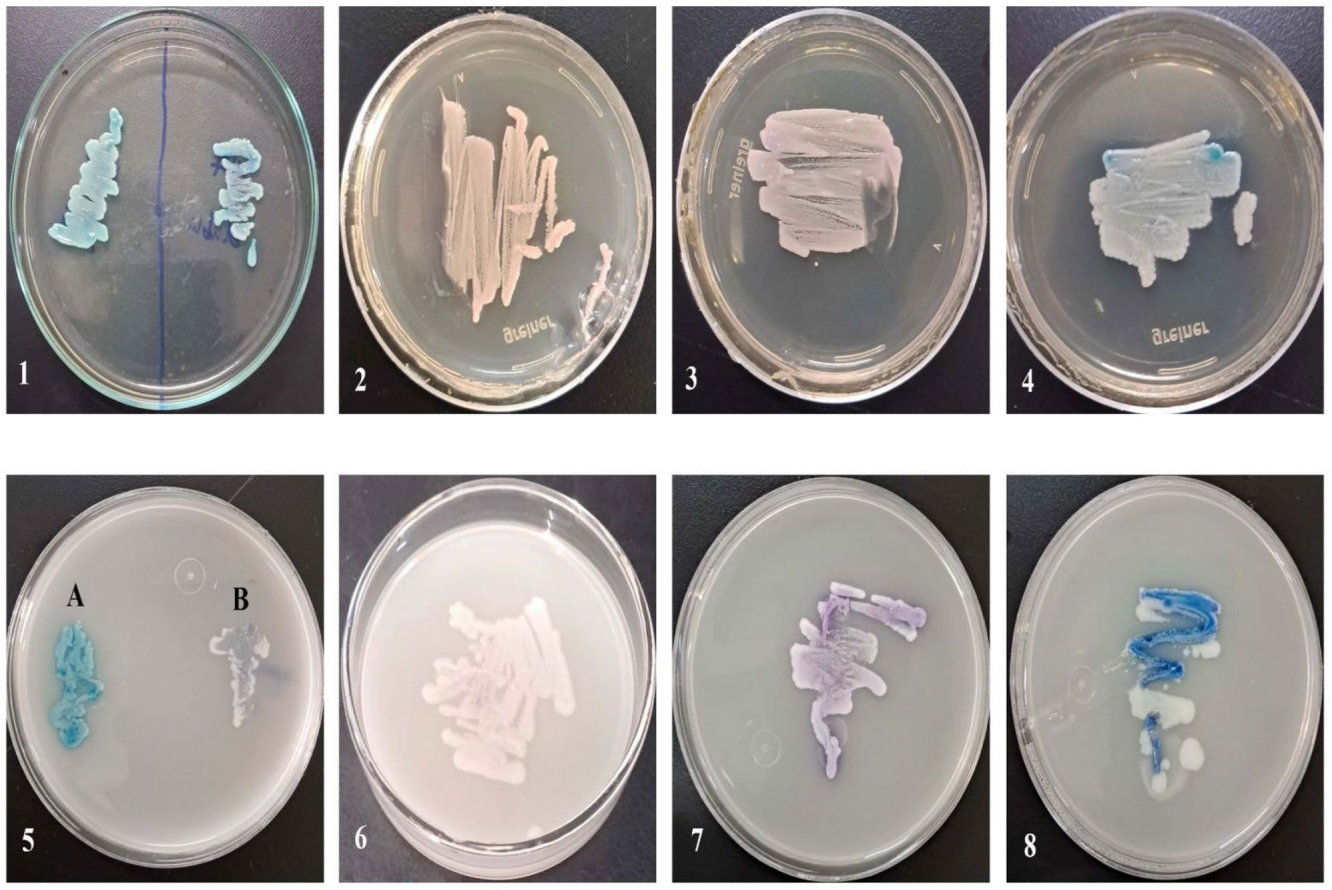
Growing isolated species from the oral cavity of intensive care units-admitted neonates on *Candida* Chromogenic Agar Medium: (1); *C. albicans*, (2); *Nakaseomyces glabratus*, (3); *Pichia kudriavzevii*, and (4); *C. tropicalis*. Also, on HiChrome™ *Candida* Differential Agar: (5 A); *C. albicans*, (5B and 6); *Nakaseomyces glabratus*, (7); *Pichia kudriavzevii* and (8); *C. tropicalis*

**Fig. 2:**
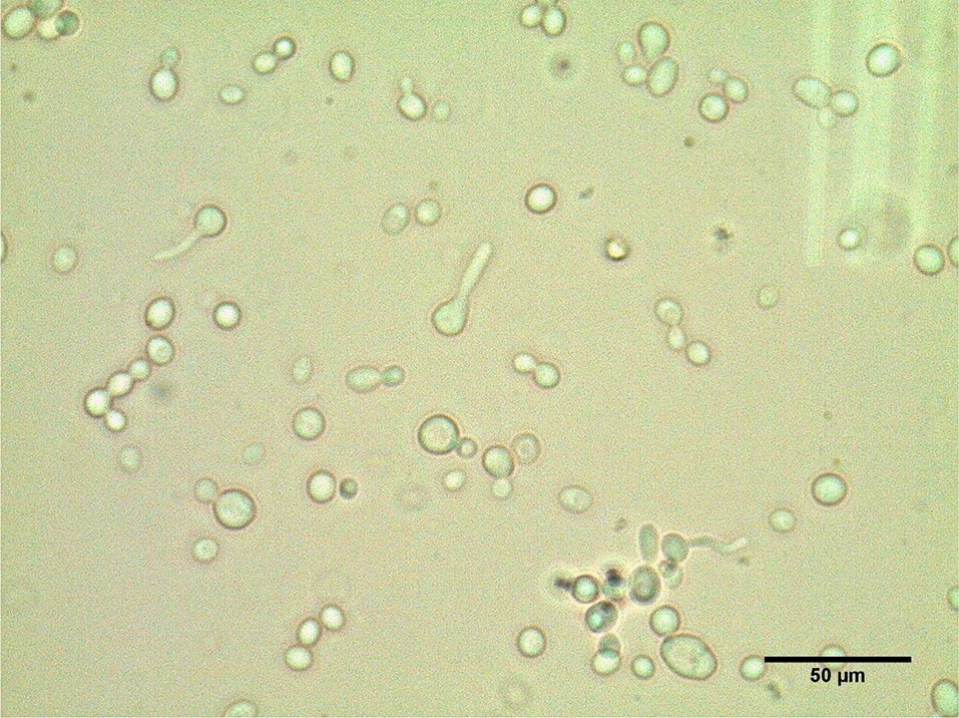
Microscopic image (40x magnification) of germ tube of isolated *C. albicans* and *C. dubliniensis* after growing species on human serum

**Fig. 3:**
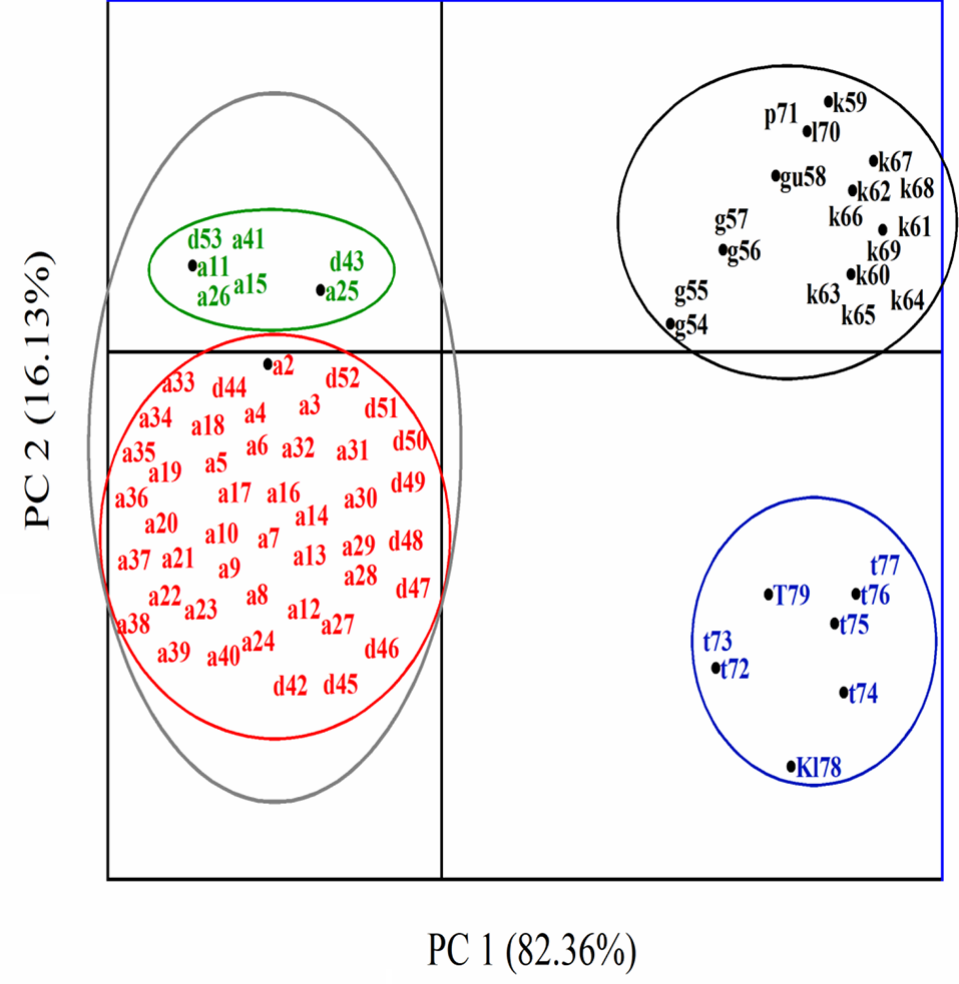
Principal-component analysis (OriginPro 2019b 64-bit.) analysis of *Candida* spp. and yeast like-structures based on culturing on *Candida* Chromogenic Agar Medium, HiChrome™ *Candida* Differential Agar and germ tube test. a: *Candida albicans*, d: *C. dubliniensis*, g: *Nakaseomyces glabratus*, gu: *Meyerozyma guilliermondii*, k: *Pichia kudriavzevii*, l: *Clavispora lusitaniae*, p: *C. parapsilosis*, t: *C. tropicalis*, K: *Kluyveromyces marxianus*, T: *Trichosporon asahii*

**Fig. 4:**
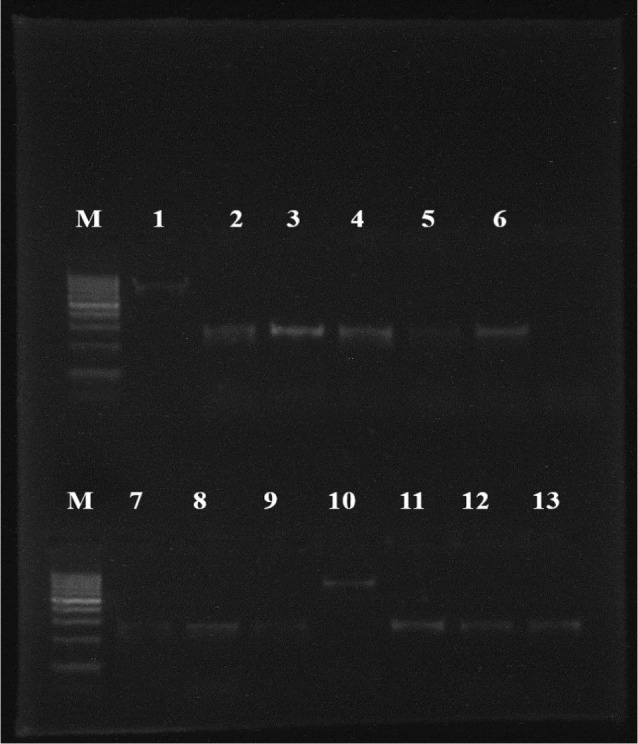
Scanning of *C*. *albicans* isolated from the oral cavity of ICU-admitted neonates with the PCR using the Gel Doc UVP Bioimaging system (GPS-8000 system). (*CALB1-CALB2*) primer used in 2,3,4,5,6,7,8,9,11,12,13 (273 bp) and (*CandF - CandR*) primer in 1,10 (850 bp) M: 100 bp molecular size marker

**Fig. 5:**
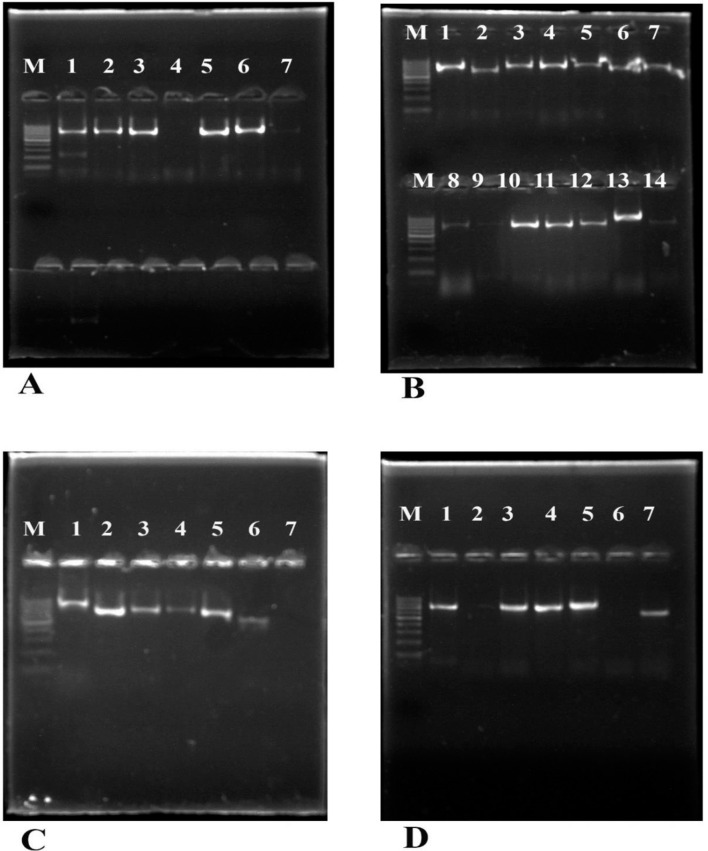
Specificity of *Candida* spp. with the PCR assay using the Gel Doc UVP Bioimaging system (GPS-8000 system). *CandF* and *CandR* primer were used, M: 100 bp molecular size marker; A: 1, 2: *C. albicans* (850 bp), 3, 5: *C. dubliniensis* (810 bp), 6: *N. glabratus* (1000 bp). B: 3, 4, 7, 9, 10, 11, 12, 14: *C*. *albicans*, 5: *C*. *dubliniensis*, 13: *N. glabratus*, 1, 6: *P. kudriavzevii* (800 bp), 2, 8: *C*. *tropicalis* (790 bp). C: 3, 4: *C*. *albicans*, 1: *N. glabratus*, 2, 5: *C*. *tropicalis*, 6: *C*. *lusitaniae* (590 bp). D: 1: *C*. *albicans*, 3, 4: *C*. *dubliniensis*, 5: *N. glabratus*, 7: *C*. *parapsilosis* (731 bp)

**Fig. 6:**
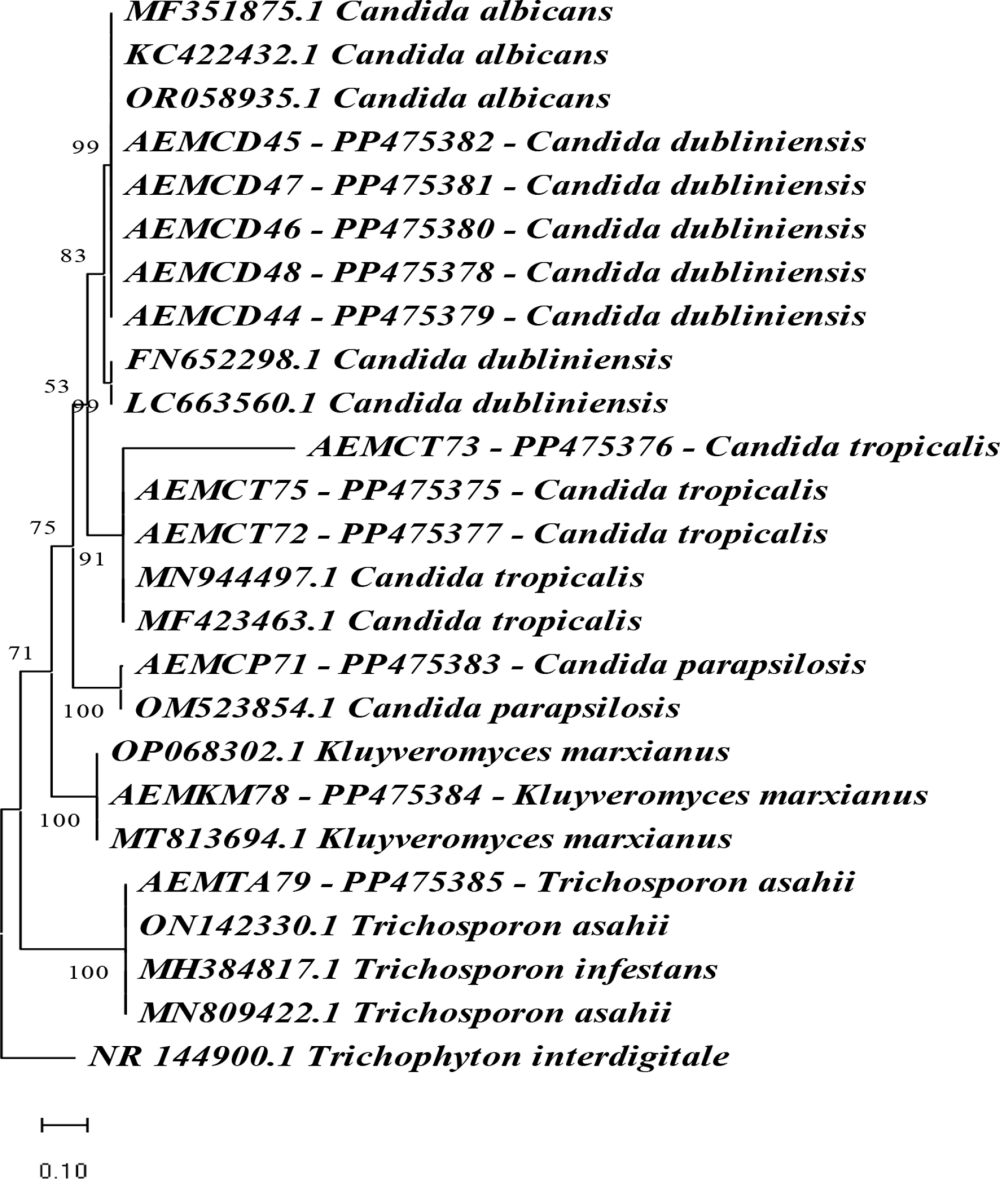
Phylogenetic tree of *Candida* spp. and yeast-like structures isolated from the oral cavity of intensive care units-admitted neonates based on ITS sequences data. The numbers above branches indicate bootstrap values that were constructed after a run of 1000 replications. Our own sequenced species were shown by codes

**Fig. 7:**
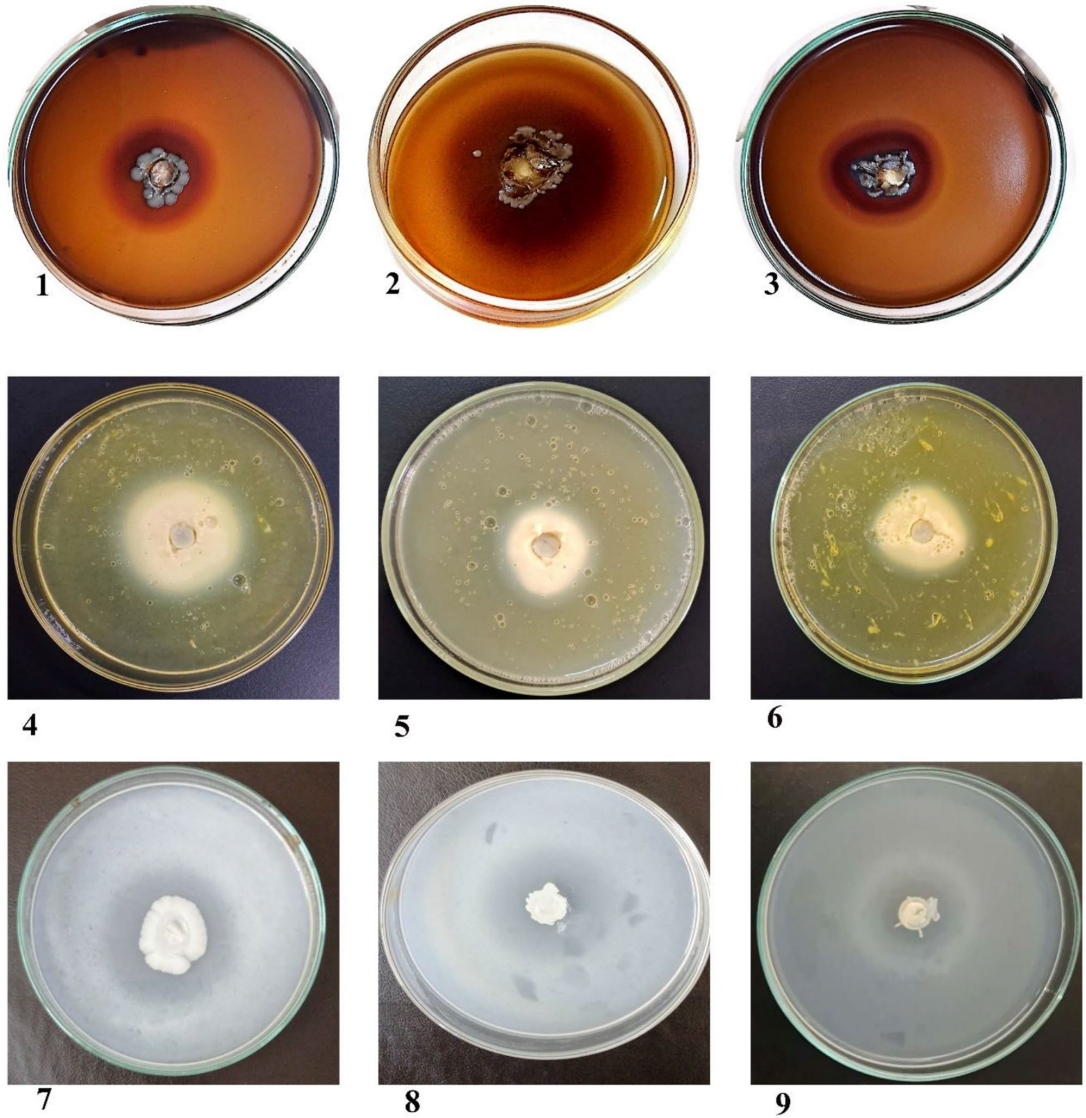
Hemolysin, phospholipase, and proteinase activities of *Candida* spp. isolated from the oral cavity of ICU-admitted neonates by growing on Sabouraud dextrose medium supplemented with 7% of human blood shows in case of hemolysin (1; *C. albicans*, 2; *N. glabratus* and 3; *P. kudriavzevii*). By growing on egg yolk agar medium for phospholipase activity (4 and 5; *C. albicans* and 6; *P. kudriavzevii*). Also, by culturing of casein hydrolysis medium for proteinase activity (7; *C. albicans*, 8; *C. tropicalis* and 9; *T. asahii*). Low (L) [1–1.50 mm], Moderate (M) [1.51–2.50 mm], High (H) [2.51–3.50 mm]

**Fig. 8:**
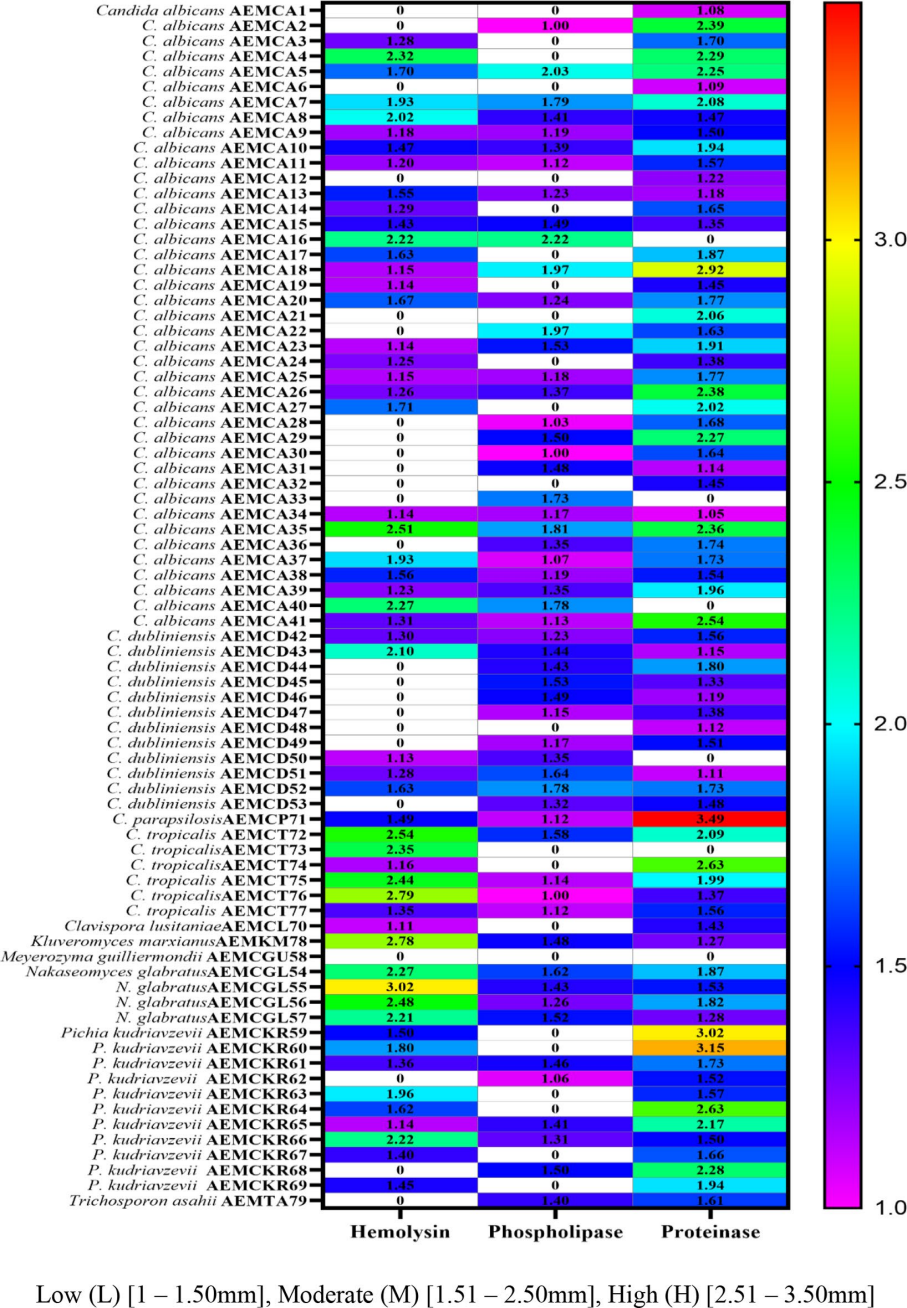
Heat map representation demonstrating the activity of hemolysin, phospholipase & proteinase enzymes secreted by the isolated yeasts using Graphpad Prism 9

Morphological characteristics of the isolated yeasts were recorded based on the color of growing isolates, on HiChrome and *Candida* Chromogenic agar media, as shown in Figs. 1 and 2 & Table 3. *C. albicans* appeared in metallic blue color on HiChrome medium (Figs. 1 and 3A), while on *Candida* Chromogenic agar, the color ranged from light green to green (Fig. 1). The number of isolates identified as *C. albicans* was 51 of the total isolates (79). The colony color of *C. glabrata/ Nakaseomyces glabratus* (AEMCGL54 and AEMCGL55) on HiChrome was white (Figs. 1, 3B and 4), while on Chromogenic agar it was pale pink, and appeared in 2 of the isolates (Figs. 1 and 5). *C. krusei/ Pichia kudriavzevii* colonies color ranged on HiChrome from light mauve to mauve, mauve with white edge, and purple blue color (Figs. 1 and 6), while on Chromogenic agar, color was pink, fuzzy pink, and light pink, which appeared in 16 of the tested isolates (Figs. 1 and 2). The color of *C. tropicalis* on HiChrome appeared dark blue, blue with white edge and blue with purple halo (Figs. 1 and 7), while on chromogenic agar ranged from blue grey to blue, blue with white edge and blue with white edge & purple halo and appeared in 5 from the total isolates (Figs. 1 and 8). Five isolates couldn’t be identified morphologically (AEMCA25, AEMCD43, AEMCT72, AEMCT73, and AEMKM78), and all isolates were subjected to molecular identification.

**Fig. 9:**
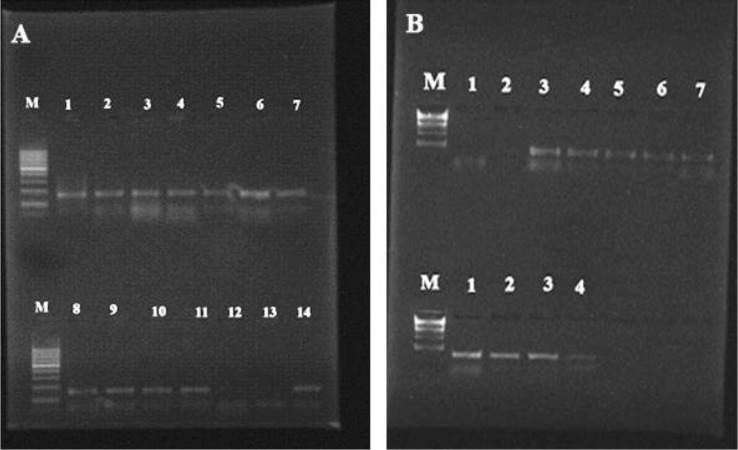
*PLB1* gene products showed by isolated strains from the oral cavity of ICU-admitted neonates at 179 pb, Lane M was a 100 bp DNA ladder, lane (1–11, 14) positive strains, (12,13) negative strains. **B**: *PLB1* and *SAP1* gene products at 179 bp and 161 bp, respectively. Lane M was a 100 bp DNA ladder, the top lanes (3, 4, 5, 6 and 7) positive strains for *PLB1* gene, and the lower lanes (1, 2, 3, and 4) positive strains for *SAP1* gene

The germ tube test was performed for all isolates. Results in Fig. 5; Table 3 revealed that the positive result appeared in all *C. albicans*, 51 of the isolates, also two isolates of AEMCA25 and AEMCD43, which couldn’t be identified by HiChrome and Chromogenic agar medium. The other isolates exhibited negative.

**Table 3:** Morphological identification of the tested isolates growing on chromogenic and HiChrome differential agar media, germ tube test results, and molecular identification by specific primer for *C. albicans* (*CALB1-CALB2*), (*CandF - CandR*) primer, and sequencing of ITS region of some species

Code	Color on Chromogenic agar	Color on HiChrome agar	germ tube test	Morphological identification	Molecular identification
AEMCA1	Light green	Metallic blue	+	*C. albicans*	*C. albicans*
AEMCA2	Light green	Metallic blue	+	*C. albicans*	*C. albicans*
AEMCA3	Light green	Metallic blue	+	*C. albicans*	*C. albicans*
AEMCA4	Light green	Metallic blue	+	*C. albicans*	*C. albicans*
AEMCA5	Light green	Metallic blue	+	*C. albicans*	*C. albicans*
AEMCA6	Light green	Metallic blue	+	*C. albicans*	*C. albicans*
AEMCA7	Light green	Metallic blue	+	*C. albicans*	*C. albicans*
AEMCA8	Light green	Metallic blue	+	*C. albicans*	*C. albicans*
AEMCA9	Light green	Metallic blue	+	*C. albicans*	*C. albicans*
AEMCA10	Light green	Metallic blue	+	*C. albicans*	*C. albicans*
AEMCA11	Green	Metallic blue	+	*C. albicans*	*C. albicans*
AEMCA12	Light green	Metallic blue	+	*C. albicans*	*C. albicans*
AEMCA13	Light green	Metallic blue	+	*C. albicans*	*C. albicans*
AEMCA14	Light green	Metallic blue	+	*C. albicans*	*C. albicans*
AEMCA15	Green	Metallic blue	+	*C. albicans*	*C. albicans*
AEMCA16	Light green	Metallic blue	+	*C. albicans*	*C. albicans*
AEMCA17	Light green	Metallic blue	+	*C. albicans*	*C. albicans*
AEMCA18	Light green	Metallic blue	+	*C. albicans*	*C. albicans*
AEMCA19	Light green	Metallic blue	+	*C. albicans*	*C. albicans*
AEMCA20	Light green	Metallic blue	+	*C. albicans*	*C. albicans*
AEMCA21	Light green	Metallic blue	+	*C. albicans*	*C. albicans*
AEMCA22	Light green	Metallic blue	+	*C. albicans*	*C. albicans*
AEMCA23	Light green	Metallic blue	+	*C. albicans*	*C. albicans*
AEMCA24	Light green	Metallic blue	+	*C. albicans*	*C. albicans*
AEMCA25	Green	Dark blue*	+	***-***	*C. albicans*
AEMCA26	Green	Metallic blue	+	*C. albicans*	*C. albicans*
AEMCA27	Light green	Metallic blue	+	*C. albicans*	*C. albicans*
AEMCA28	Light green	Metallic blue	+	*C. albicans*	*C. albicans*
AEMCA29	Light green	Metallic blue	+	*C. albicans*	*C. albicans*
AEMCA30	Light green	Metallic blue	+	*C. albicans*	*C. albicans*
AEMCA31	Light green	Metallic blue	+	*C. albicans*	*C. albicans*
AEMCA32	Light green	Metallic blue	+	*C. albicans*	*C. albicans*
AEMCA33	Light green	Metallic blue	+	*C. albicans*	*C. albicans*
AEMCA34	Light green	Metallic blue	+	*C. albicans*	*C. albicans*
AEMCA35	Light green	Metallic blue	+	*C. albicans*	*C. albicans*
AEMCA36	Light green	Metallic blue	+	*C. albicans*	*C. albicans*
AEMCA37	Light green	Metallic blue	+	*C. albicans*	*C. albicans*
AEMCA38	Light green	Metallic blue	+	*C. albicans*	*C. albicans*
AEMCA39	Light green	Metallic blue	+	*C. albicans*	*C. albicans*
AEMCA40	Light green	Metallic blue	+	*C. albicans*	*C. albicans*
AEMCA41	Green	Metallic blue	+	*C. albicans*	*C. albicans*
AEMCD42	Light green	Metallic blue	+	*C. albicans*	*C. dubliniensis*
AEMCD43	Green	Dark blue*	+	***-***	*C. dubliniensis*
AEMCD44	Light green	Metallic blue	+	*C. albicans*	*C. dubliniensis*
AEMCD45	Light green	Metallic blue	+	*C. albicans*	*C. dubliniensis*
AEMCD46	Light green	Metallic blue	+	*C. albicans*	*C. dubliniensis*
AEMCD47	Light green	Metallic blue	+	*C. albicans*	*C. dubliniensis*
AEMCD48	Light green	Metallic blue	+	*C. albicans*	*C. dubliniensis*
AEMCD49	Light green	Metallic blue	+	*C. albicans*	*C. dubliniensis*
AEMCD50	Light green	Metallic blue	+	*C. albicans*	*C. dubliniensis*
AEMCD51	Light green	Metallic blue	+	*C. albicans*	*C. dubliniensis*
AEMCD52	Light green	Metallic blue	+	*C. albicans*	*C. dubliniensis*
AEMCD53	Green	Metallic blue	+	*C. albicans*	*C. dubliniensis*
AEMCGL54	Light pink	White	-	*C. glabrata*	*N. glabratus*
AEMCGL55	Light pink	White	-	*C. glabrata*	*N. glabratus*
AEMCGL56	Pink	Light mauve	-	*C. krusei*	*N. glabratus*
AEMCGL57	Pink	Light mauve	-	*C. krusei*	*N. glabratus*
AEMCGU58	Fuzzy pink	Mauve	-	*C. krusei*	*M. guilliermondii*
AEMCKR59	Pink	Purple blue	-	*C. krusei*	*P. kudriavzevii*
AEMCKR60	Pink	Mauve	-	*C. krusei*	*P. kudriavzevii*
AEMCKR61	Pink	Mauve with white edge	-	*C. krusei*	*P. kudriavzevii*
AEMCKR62	Fuzzy pink	Mauve with white edge	-	*C. krusei*	*P. kudriavzevii*
AEMCKR63	Pink	Mauve	-	*C. krusei*	*P. kudriavzevii*
AEMCKR64	Pink	Mauve	-	*C. krusei*	*P. kudriavzevii*
AEMCKR65	Pink	Mauve	-	*C. krusei*	*P. kudriavzevii*
AEMCKR66	Fuzzy pink	Mauve with white edge	-	*C. krusei*	*P. kudriavzevii*
AEMCKR67	Fuzzy pink	Purple blue	-	*C. krusei*	*P. kudriavzevii*
AEMCKR68	Fuzzy pink	Mauve with white edge	-	*C. krusei*	*P. kudriavzevii*
AEMCKR69	Pink	Mauve with white edge	-	*C. krusei*	*P. kudriavzevii*
AEMCL70	Light pink	Mauve with white edge	-	*C. krusei*	*C. lusitaniae*
AEMCP71	Light pink	Mauve with white edge	-	*C. krusei*	*C. parapsilosis*
AEMCT72	Blue	Metallic blue*	-	**-**	*C. tropicalis*
AEMCT73	Blue	Metallic blue*	-	***-***	*C. tropicalis*
AEMCT74	Blue with white edge & purple halo	Dark blue	-	*C. tropicalis*	*C. tropicalis*
AEMCT75	Blue grey	Blue with purple halo	-	*C. tropicalis*	*C. tropicalis*
AEMCT76	Blue grey	Blue with white edge	-	*C. tropicalis*	*C. tropicalis*
AEMCT77	Blue grey	Blue with white edge	-	*C. tropicalis*	. *C. tropicalis*
AEMKM78	Blue with white edge	Metallic blue*	-	*-*	*K. marxianus*
AEMTA79	Blue	Dark blue	-	*C. tropicalis*	*T. asahii*

(*) for sample we couldn’t identify on Hichrome and Chromogenic agar

### PCA-based classification of morphological characteristics in the isolated strains

By looking at the left side of Fig. 2, fifty-three isolates inside the grey circle exhibited a positive germ tube test, and most of them were identified as *C. albicans*. The red circle contained isolates that showed a light green color on Chromogenic medium and a metallic blue color in HiChrome. Isolates in green circles showed green color on Chromogenic medium, and metallic blue to dark blue when grown on HiChrome. On the right side, a black circle containing eighteen isolates gave shades of pink color on the Chromogenic medium and negative germ tube test. Also appeared with a white color, a blue-purple color, a pale mauve, and mauve with a white edge to mauve color on HiChrome medium. The blue circle showed the isolates with shades of blue color, whether on Chromogenic or HiChrome medium, and a negative germ tube test. This interference between some isolates in colors made us resort to molecular identification.

### Genetic profile of the isolated samples using different molecular techniques

For accurate identification, the tested isolates were subjected to molecular characterization. Firstly, isolates were tested with the specific primer (*CALB1- CALB2*) for detecting *C. albicans.* Only 41 isolates (AEMCA1 to AEMCA41) produced band at 273 bp, 40 isolates identified as *C. albicans* on HiChrome and *Candida* Chromogenic agar media and one isolate from we couldn’t identify morphologically (AEMCA25), germ tube test was also positive, so this isolate was identified as *C. albicans* this was shown in (Table 3; Fig. 8).

Twelve isolates (AEMCD42 to AEMCD53) failed to exhibit a band by (*CALB1-CALB2*) primer. Eleven were identified as *C. albicans* by macroscopic and microscopic criteria, and one isolate AEMCD43 (didn’t identify via the two media), so confirmed as *C. dubliniensis* by using sequencing; also, the germ tube test was positive. The previous 12 isolates were tested with (CandF - CandR) primer, and they showed a band at 810 bp, which couldn’t distinguish from 850 bp for *C. albicans*, so we couldn’t differentiate them. Thus, five isolates from them were subjected to the sequence technique (AEMCD44, AEMCD45, AEMCD46, AEMCD47, and AEMCD48), as representative isolates to confirm our identification.

The rest of the species were identified using (*CandF - CandR*) primers to identify eight species of *Candida* based on the analysis of specific regions such as insertions, deletions of different nucleotides, or regions with specific sequences. 4 isolates showed a band at 1000 bp (AEMCGL54 to AEMCGL57), identified as *C. glabrata*/ *Nakaseomyces glabratus*. One isolate *C. guilliermondii*/*Meyerozyma guilliermondii* (AEMCGU58) exhibited a band at 1100 bp. Eleven isolates showed a band at 800 bp, so they were identified as *C. krusei/Pichia kudriavzevii*, one isolate, *C. lusitaniae*/*Clavispora lusitaniae* (AEMCL70) showed a band at 590 bp, another one *C. parapsilosis* failed to identify by (*CandF-CandR*) primer and identified by sequencing (AEMCP71), 6 of isolates showed a band at 790 bp, so it was identified as *C. tropicalis* (Fig. 4), and we confirmed our identification by sequencing of three isolates AEMCT72, AEMCT73, and AEMCT75, as representative isolates and AEMKM78 and AEMTA79 were identified by sequencing as *Kluyveromyces maxianus* and *Trichosporon asahii*, respectively. Results are clearly illustrated in Fig. 3.

### Phylogenetic analysis of the identified isolates

Figure 4 shows that eleven isolates were chosen to confirm the identification. Firstly, the amplified sequences were subjected to the GenBank database, and then the strain sequences, which were obtained by using the ITS region gene, were compared with other yeast sequences introduced in the GenBank database. The score of the highest value of the sequence was filtered from the databases and aligned with ITS sequences of the *Candida* spp. and some yeast-like structures. Mega X software was applied to study the genetic relatedness of 11 strains. Results from BLAST revealed that *Candida albicans*, *C. dubliniensis*, *C. parapsilosis*,* C. tropicalis*, and *Kluveromyces marxianus* belong to the phylum Ascomycota; order Saccharomycetales. *C. dubliniensis* strains (AEMCD45-PP475382, AEMCD47-PP475381, AEMCD46- PP475380, AEMCD48- PP475378, and AEMCA44- PP475379) formed a distinct subclade with *C. albicans* MF351875.1, *C. albicans* KC422432.1 and *C. albicans* OR058935.1 and supported by bootstrap value of 99%. All the previous *C. albicans* & *C. dubliniensis* were clustered with *C. dubliniensis* FN652298.1 and *C. dubliniensis* LC663560.1 obtained from GenBank by a bootstrap value of 83%. But according to our identification of strains by specific primer for *C. albicans* and by (*CandF-CandR*) primer which showed that strains with code AEMCD45, AEMCD47, AEMCD46, AEMCD48, and AEMCA44 were identified as *C. dubliniensis*. *C. tropicalis* (AEMCT73- PP475376, AEMCT75- PP475375 & AEMCT72- PP475377) were grouped in the same clade with C. *tropicalis* MN944497.1 and *C. tropicalis* MF423463.1 that were recorded in GenBank by 91% bootstrap value. *C. parapsilosis* AEMCP71-PP475383.1 was in the same subclade as *C. parapsilosis* OM523854.1 with a bootstrap value of 100%. *Kluyveromyces marxianus* AEMKM78-PP475384 was grouped with *Kluyveromyces marxianus* OP068302.1 and *Kluyveromyces marxianus* MT813694.1 and supported by 100% bootstrap value. *Trichosporon asahii* AEMTA79-PP475385 belongs to division: Basidiomycota, class: Tremellomycetes, and order: Trichosporonales was in the same subclade with *Trichosporon asahii* ON142330.1, *Trichosporon infestans* MH384817.1 and *Trichosporon asahii* MN809422.1 with bootstrap value of 100%. Finally, *Trichophyton interdigitale* NR144900.1 was settled at the end of the phylogenic tree as an out-group.

### Virulence characteristics of invasive isolates

Enzymatic activity of the isolated samples showed that thirty-six isolates were active producers of the hemolysin, phospholipase, and proteinase enzymes with variable efficiencies encompassing: nineteen isolates of *C. albicans*, four isolates of *C. dubliniensis*, four isolates of *N. glabratus*, three isolates of *P. kudriavzevii*, one strain of *C. parapsilosis*, four isolates of *C. tropicalis*, and one isolate of *K. marxianus* as illustrated in Table 4; Figs. 6 and 7.

**Table 4:** Statistical analysis of hemolysin (H), phospholipase (ph.), proteinase (Pr) activity of the isolated species using SPSS16 (One way ANOVA), survey of *SAP1* and *PLB1* genes related to phospholipase and proteinase secretion

Code	Gender	Species	Mean (H) ± STD.	Mean (ph.) ± STD.	*PLB1*	Mean (Pr) ± STD.	*SAP1*
AEMCA1	Female	*Candida albicans*	-	-	+ve	1.08*±0.14(L)	+ve
AEMCA2	Male	*C. albicans*	-	1*±0.00(L)	+ve	2.39*±0.28(M)	+ve
AEMCA3	Male	*C. albicans*	1.28*±0.10 (L)	-	+ve	1.70*±0.61(M)	+ve
AEMCA4	Female	*C. albicans*	2.32*±0.75 (M)	-	+ve	2.29*±0.13(M)	+ve
AEMCA5	Male	*C. albicans*	1.70*±0.43(M)	2.03*±0.25(M)	+ve	2.25*±0.19(M)	-
AEMCA6	Male	*C. albicans*	-	-	+ve	1.09*±0.16(L)	-
AEMCA7	Male	*C. albicans*	1.93*±0.23(M)	1.79*±0.09(M)	+ve	2.08*±1.01(M)	+ve
AEMCA8	Female	*C. albicans*	2.02*±0.30(M)	1.41*±0.42(L)	*-*	1.47*±0.12(L)	+ve
AEMCA9	Female	*C. albicans*	1.18*±0.16(L)	1.19*±0.32(L)	+ve	1.50*±0.26(L)	-
AEMCA10	Female	*C. albicans*	1.47*±0.25(L)	1.39*±0.53(L)	+ve	1.94*±0.19(M)	+ve
AEMCA11	Female	*C. albicans*	1.20*±0.15(L)	1.12*±0.16(L)	+ve	1.57*±0.23(M)	+ve
AEMCA12	Male	*C. albicans*	-	-	+ve	1.22*±0.39(L)	+ve
AEMCA13	Male	*C. albicans*	1.55*±0.64(M)	1.23*±0.22(L)	+ve	1.18*±0.11(L)	-
AEMCA14	Male	*C. albicans*	1.29*±0.18(L)	-	*-*	1.65*±0.08(M)	+ve
AEMCA15	Male	*C. albicans*	1.43*±0.16(L)	1.49*±0.43(L)	+ve	1.35*±0.03(L)	-
AEMCA16	Female	*C. albicans*	2.22*±0.12(M)	2.22*±1.46(M)	+ve	-	-
AEMCA17	Female	*C. albicans*	1.63*±0.24(M)	-	+ve	1.87*±0.34(M)	-
AEMCA18	Male	*C. albicans*	1.15*±0.06(L)	1.97*±1.06(M)	+ve	2.92*±0.09(H)	+ve
AEMCA19	Female	*C. albicans*	1.14*±0.19(L)	-	*-*	1.45*±0.23(L)	-
AEMCA20	Male	*C. albicans*	1.67*±0.23(M)	1.24*±0.12(L)	+ve	1.77*±0.31(M)	+ve
AEMCA21	Male	*C. albicans*	-	-	+ve	2.06*±0.44(M)	-
AEMCA22	Male	*C. albicans*	-	1.97*±0.15(M)	+ve	1.63*±0.78(M)	-
AEMCA23	Male	*C. albicans*	1.14*±0.07(L)	1.53*±0.18(M)	+ve	1.91*±0.40(M)	+ve
AEMCA24	Male	*C. albicans*	1.25*±0.15(L)	-	+ve	1.38*±0.02(L)	+ve
AEMCA25	Male	*C. albicans*	1.15*±0.07(L)	1.18*±0.31(L)	+ve	1.77*±0.17(M)	+ve
AEMCA26	Female	*C. albicans*	1.26*±0.04(L)	1.37*±0.34(L)	+ve	2.38*±0.30(M)	-
AEMCA27	Female	*C. albicans*	1.71*±0.52(M)	-	+ve	2.02*±0.30(M)	+ve
AEMCA28	Female	*C. albicans*	-	1.03*±0.06(L)	+ve	1.68*±0.31(M)	-
AEMCA29	Male	*C. albicans*	-	1.50*±0.50(L)	+ve	2.27*±0.45(M)	+ve
AEMCA30	Male	*C. albicans*	-	1*±0.00(L)	+ve	1.64*±0.22(M)	+ve
AEMCA31	Male	*C. albicans*	-	1.48*±0.48(L)	+ve	1.14*±0.13(L)	-
AEMCA32	Female	*C. albicans*	-	-	+ve	1.45*±0.24(L)	+ve
AEMCA33	Female	*C. albicans*	-	1.73*±0.74(M)	+ve	-	+ve
AEMCA34	Female	*C. albicans*	1.14*±0.09(L)	1.17*±0.15(L)	+ve	1.05*±0.09(L)	+ve
AEMCA35	Male	*C. albicans*	2.51*±0.21(H)	1.81*±0.15(M)	+ve	2.36*±0.49(M)	+ve
AEMCA36	Male	*C. albicans*	-	1.35*±0.31(L)	+ve	1.74*±0.61(M)	+ve
AEMCA37	Female	*C. albicans*	1.93*±1.02(M)	1.07*±0.12(L)	+ve	1.73*±0.23(M)	+ve
AEMCA38	Male	*C. albicans*	1.56*±0.19(M)	1.19*±0.07(L)	+ve	1.54*±0.18(M)	+ve
AEMCA39	Female	*C. albicans*	1.23*±0.19(L)	1.35*±0.61(L)	+ve	1.96*±1.49(M)	+ve
AEMCA40	Male	*C. albicans*	2.27*±0.25(M)	1.78*±0.34(M)	+ve	-	-
AEMCA41	Male	*C. albicans*	1.31*±0.01(L)	1.13*±0.23(L)	+ve	2.54*±0.67(H)	+ve
AEMCD42	Male	*C. dubliniensis*	1.30*±0.28(L)	1.23*±0.07(L)	+ve	1.56*±0.38(M)	+ve
AEMCD43	Female	*C. dubliniensis*	2.10*±0.40(M)	1.44*±0.38(L)	+ve	1.15*±0.10(L)	-
AEMCD44	Female	*C. dubliniensis*	-	1.43*±0.38(L)	+ve	1.80*±0.04(M)	-
AEMCD45	Male	*C. dubliniensis*	-	1.53*±0.53(M)	+ve	1.33*±0.00(L)	-
AEMCD46	Female	*C. dubliniensis*	-	1.49*±0.61(L)	+ve	1.19*±0.09(L)	-
AEMCD47	Female	*C. dubliniensis*	-	1.15 ± 0.15(L)	*-*	1.38*±0.06(L)	-
AEMCD48	Male	*C. dubliniensis*	-	-	*-*	1.12*±0.13(L)	-
AEMCD49	Male	*C. dubliniensis*	-	1.17*±0.14(L)	+ve	1.51*±0.09(M)	-
AEMCD50	Female	*C. dubliniensis*	1.13*±0.11(L)	1.35*±0.10(L)	*-*	-	-
AEMCD51	Male	*C. dubliniensis*	1.28*±0.05(L)	1.64*±0.41(M)	*-*	1.11*±0.05(L)	+ve
AEMCD52	Male	*C. dubliniensis*	1.63*±0.49(M)	1.78*±0.92(M)	*-*	1.73*±0.11(M)	-
AEMCD53	Male	*C. dubliniensis*	-	1.32*±0.45(L)	*-*	1.48*±0.11(L)	-
AEMCGL54	Female	*N. glabratus*	2.27*±0.59(M)	1.62*±0.27(M)	+ve	1.87*±0.21(M)	+ve
AEMCGL55	Female	*N. glabratus*	3.02*±0.29(H)	1.43*±0.16(L)	+ve	1.53*±0.26(M)	-
AEMCGL56	Female	*N. glabratus*	2.48*±0.30(M)	1.26*±0.26(L)	+ve	1.82*±1.02(M)	+ve
AEMCGL57	Male	*N. glabratus*	2.21*±0.08(M)	1.52*±0.16(M)	+ve	1.28*±0.29(L)	-
AEMCGU58	Male	*M. guilliermondii*	-	-	+ve	-	-
AEMCKR59	Male	*P. kudriavzevii*	1.50*±0.37(L)	-	+ve	3.02*±0.29(H)	-
AEMCKR60	Female	*P. kudriavzevii*	1.80*±0.17(M)	-	+ve	3.15*±1.01(H)	-
AEMCKR61	Male	*P. kudriavzevii*	1.36*±0.31(L)	1.46*±0.42(L)	*-*	1.73*±0.48(M)	-
AEMCKR62	Male	*P. kudriavzevii*	-	1.06*±0.07(L)	*-*	1.52*±0.41(M)	-
AEMCKR63	Male	*P. kudriavzevii*	1.96*±0.51(M)	-	+ve	1.57*±0.59(M)	-
AEMCKR64	Female	*P. kudriavzevii*	1.62*±0.10(M)	-	+ve	2.63*±0.47(H)	-
AEMCKR65	Female	*P. kudriavzevii*	1.14*±0.09(L)	1.41*±0.70(L)	+ve	2.17*±0.29(M)	-
AEMCKR66	Female	*P. kudriavzevii*	2.22*±0.39(M)	1.31*±0.53(L)	+ve	1.50*±0.18(L)	-
AEMCKR67	Male	*P. kudriavzevii*	1.40*±0.21(L)	-	+ve	1.66*±0.20(M)	-
AEMCKR68	Male	*P. kudriavzevii*	-	1.50*±0.81(L)	+ve	2.28*±0.63(M)	-
AEMCKR69	Male	*P. kudriavzevii*	1.45*±0.14(L)	-	*-*	1.94*±0.25(M)	-
AEMCL70	Male	*C. luistaniae*	1.11*±0.05(L)	-	+ve	1.43*±0.12(L)	-
AEMCP71	Female	*C. parapsilosis*	1.49*±0.12(L)	1.12*±0.20(L)	+ve	3.49*±0.55(H)	-
AEMCT72	Female	*C. tropicalis*	2.54*±0.05(H)	1.58*±0.33(M)	*-*	2.09*±0.73(M)	-
AEMCT73	Female	*C. tropicalis*	2.35*±0.42(M)	-	+ve	-	-
AEMCT74	Male	*C. tropicalis*	1.16*±0.06(L)	-	+ve	2.63*±0.74(H)	-
AEMCT75	Female	*C. tropicalis*	2.44*±0.10(M)	1.14*±0.19(L)	+ve	1.99*±0.39(M)	+ve
AEMCT76	Male	*C. tropicalis*	2.79*±0.43(H)	1*±0.00(L)	+ve	1.37*±0.34(L)	+ve
AEMCT77	Male	*C. tropicalis*	1.35*±0.07(L)	1.12*±0.10(L)	*-*	1.56*±0.05(M)	-
AEMKM78	Female	*K. marxianus*	2.78*±0.05(H)	1.48*±0.33(L)	+ve	1.27*±0.08(L)	-
AEMCTA79	Male	*T. asahii*	-	1.40*±0.46(L)	*-*	1.61*±0.29(M)	-

STD. means standard deviation. Low (L) [1–1.50 mm], Moderate (M) [1.51–2.50 mm], High (H) [2.51–3.50 mm]

Hemolysin activity appeared in fifty-five (69.6%) out of the total isolates; high production (H) exhibited by five isolates (9.1%), moderate secretion (M) clearly appeared in twenty-three (41.8%), and low result (L) was shown by twenty-seven isolates (49.1%) out of positive isolates (Table 4; Fig. 7).

Phospholipase activity appeared in fifty-six (70.9%) of the total isolates; high production (H) of phospholipase enzyme didn’t exhibit by the tested isolates, moderate secretion (M) was displayed by fifteen (26.8%), and low result (L) was shown by forty-one (73.2%) out of the positive isolates (Table 4; Fig. 7).

Proteinase activity exhibited seventy-three (92.4%) of the tested isolates; high secretion (H) of proteinase appeared within seven (9.6%), moderate result (M) appeared in forty-two (57.5%), and low result (L) appeared in twenty-four isolates (32.9%) out of the secreted isolates (Table 4; Fig. 7).

*C. albicans* exhibited a significant secretion of the three tested enzymes, with 19 isolates producing hemolysin, 29 secreting phospholipase, and 33 producing proteinase (Table 4; Fig. 7).

High measuring dark zones of the secreted hemolysin were exhibited by five isolates (9.1%); two isolates were *C. tropicalis* (AEMCT72, and AEMCT76), and one isolate of *C. albicans* (AEMCA35), *C. glabrata* (AEMCGL55), and *Kluyveromyces marxianus* (AEMKM78). phospholipase high production (H) didn’t exhibit by the tested isolates, but moderate secretion (M) of phospholipase enzyme displayed by fifteen (26.8%) out of the positive isolates; nine of them were *C*. *albicans* (AEMCA5, AEMCA7, AEMCA16, AEMCA18, AEMCA22, AEMCA23, AEMCA33, AEMCA35, and AEMCA40), three of *C*. *dubliniensis* (AEMCD45, AEMCD51, and AEMCD52), two of *C. glabrata* (AEMCGL54 and AEMCGL57), and only one isolate of *C. tropicalis* (AEMCT72). The higher clear zone formed via proteinase secretion appeared within seven (9.6%) of the productive isolates; two of *C. albicans* (AEMCA18 and AEMCA41), three of *C. krusei* (AEMCKR59, AEMCKR60, and AEMCKR64), one was *C. parapsilosis* (AEMCP71), and another one of *C. tropicalis* (AEMCT74). For details, return to (Figs. 6 and 7, & Table 4).

Distribution of virulence gene among the isolated strains revealed that sixty-four isolates from tested yeast were positive for the presence of phospholipase B1 (*PLB1*) gene, 38 of these isolates were *C. albicans*, 5 strains of *C. dubliniensis*, four isolates of *N. glabratus*, one isolate of *M. guilliermondii* (AEMCGU58), 8 isolates of *P. kudriavzevii*, one strain of *C. lusitaniae* (AEMCL70), one strain of *C. parapsilosis* (AEMCP71), four strains of *C. tropicalis* and one isolate of *K. marxianus* (AEMKM78) as shown in Fig. 9; Table 4.

*PLB1* is closely related to phospholipase enzyme secretion as it is responsible for phospholipase B1 secretion, which represents a type of phospholipase. The results showed that forty-five (57%) were positive for the presence of *PLB1* gene and exhibited phospholipase enzyme. 28 strains (35.4%) of *C. albicans*, 6 isolates (7.6%) of *C. dubliniensis*, 4 strains (5.1%) of *N. glabratus*, 3 isolates (3.8%) of *P. kudriavzevii*, one isolate (1.3%) of *C. parapsilosis*, 2 isolates (2.5%) of *C*. *tropicalis*, and one isolate (1.3%) of *K. marxianus* (AEMKM78). Eleven strains (13.9%) secreted phospholipase enzyme but didn’t show *PLB1* gene. One isolate of *C. albicans* (AEMCA8), 5 isolates of *C. dubliniensis* (AEMCD48, AEMCD51, AEMCD52, AEMCD53, and AEMCD54), 2 isolates of *P. kudriavzevii* (AEMCKR61 and AEMCKR62), 2 isolates of *C*. *tropicalis* (AEMCT72 and AEMCT77) and one strain of *Trichosporon asahii* (AEMTA79).

Nineteen isolates (24.1%) showed positive of *PLB1* gene but didn’t secrete phospholipase enzyme, 10 isolates (12.7%) of *C. albicans* (AEMCA1, AEMCA3, AEMCA4, AEMCA6, AEMCA12, AEMCA17, AEMCA21, AEMCA24, AEMCA27, and AEMCA32), one strain (1.3%) of *M. guilliermondii* (AEMCGU58), 5 isolates (6.3%) of *P. kudriavzevii* (AEMCKR59, AEMCKR60, AEMCKR63, AEMCKR64 and AEMCKR67), one isolate (1.3%) of *C. lusitaniae* (AEMCL70) and two isolates (2.5%) of *C*. *tropicalis* (AEMCT73 and AEMCT74). Four isolates (5.1%) neither expressed *PLB1* gene nor secreted phospholipase enzyme, 2 isolates of *C. albicans* (AEMCA14 and AEMCA19), one isolate of *C. dubliniensis* (AEMCD48), and one *P. kudriavzevii* (AEMCKR69). Thirty-three isolates (41.8%) from the isolated yeast were positive for the presence of *SAP1* gene. 27 (34.2%) of these isolates were *C. albicans*, 2 (2.5%) of *C. dubliniensis*, 2 (2.5%) of *N. glabratus* and 2 (2.5%) of *C. tropicalis*.

*SAP1* is related to aspartic protease 1 production, which is a type of sap that represents one of those responsible for proteinase secretion. The results showed that thirty-two isolates expressed *SAP1* gene and secreted the proteinase enzyme. 26 isolates of *C. albicans*, 2 isolates of *C. dubliniensis*, 2 strains of *N. glabratus* and 2 isolates of *C. tropicalis*. Forty-one isolates secreted proteinase enzyme and didn’t express the *SAP1* gene. 12 isolates (15.2%) of *C. albicans*, 9 isolates (11.4%) of *C. dubliniensis*, two isolates (2.5%) of *N. glabratus*, 11 isolates (13.9%) of *P. kudriavzevii*, one isolate (1.3%) of *C. lusitaniae*, one strain (1.3%) of *C. parapsilosi*s, 3 isolates (3.8%) of *C. tropicalis*, one strain (1.3%) of *K. maxianus* and one strain (1.3%) of *T. asahii*.

One isolate (1.3%) didn’t secrete proteinase enzyme but expressed *SAP1* gene, *C. albicans* (AEMCA33). Five isolates expressed neither *SAP1* gene nor secreted proteinase enzyme, 2 isolates of *C. albicans* (AEMCA16 and AEMCA40), one isolate of *C. dubliniensis* (AEMCD50), one isolate of *M. guilliermondii*, (AEMCGU58), and one isolate of *C. tropicalis* (AEMCT73). This was illustrated in Fig. 9; Table 4.

### Distribution of yeast species and their virulence factors among male and female isolates

The distribution of thrush in Table 4 shows a total of 45 male and 34 female isolates. Among them, *C. albicans* was identified in 24 male and 17 female samples, while *C. dubliniensis* appeared in 7 male and 5 female cases. *N. glabratus* was found in a single male sample and 3 female samples. *P. kudriavzevii* was present in 7 male and 5 female samples, whereas *Candida tropicalis* was equally distributed with 3 male and 3 female occurrences. *C. lusitaniae* and *T. asahii* were exclusively found in male samples, while *C. parapsilosis* and *K. marxianus* appeared only in female samples (Table 4).

The three types of enzymes were secreted equally by both males and females, with a balanced distribution of 18 males and 18 females. Out of 55 isolates identified as active producers of hemolysin, 28 were from males and 27 were from females. Phospholipase activity was observed in 31 male samples and 25 female samples, out of which 24 males and 21 females showed *PLB1* gene. Proteinase activity was detected in 43 samples of males and 30 samples of females; among them, 19 males and 9 females exhibited the *SAP1* gene. As illustrated in Table 4.

## Discussion

Our study aims to assess the prevalence of neonatal oral thrush in Qena Governorate, Egypt. The collected isolates were subjected to laboratory experiments for accurate identification and virulence analysis. A total of 79 isolates were obtained, comprising 45 from male samples and 34 from females. The disparity in the number from males compared to females could be attributed to the highest number of the male samples collected during our analysis. Kumar [51] suggested that thrush affects males and females at an equal rate.

The collected isolates were identified according to their morphological and genetic characteristics as shown in Table 3. Low birth weight and prematurity can increase the likelihood of oral thrush in newborns admitted to ICU, this agrees with Dermitzaki et al. [52], preterm and low-birth-weight neonates are the most vulnerable to the disease’s dissemination. Antimicrobial peptides play a crucial role in the body’s natural immune defenses. In premature newborns, their level is typically low, making them more vulnerable to infections [53]. Also, antibiotics, central catheter, and endotracheal tube, along with the healthcare setting, have been strictly associated with a higher risk of invasive candidiasis as described by Benjamin et al. [54].

Recently, several species of *Candida* have been renamed with different genera, thus these genera don’t consider as a causative agent of candidiasis anymore, however they share *Candida* spp. in many characteristics such as epidemiology and management [55] *Candida* belongs to Kingdom: Fungi, Division: Ascomycota, Class: Saccharomycetes, Order: Saccharomycetales [56] Family: Debaryomycetaceae comprising several species such as (*C. albicans*, *C. dubliniensis*, *C. guilliermondii* (*Meyerozyma guilliermondii*), *C. parapsilosis*, *C. tropicalis*…etc.). *C. glabrata* (*Nakaseomyces glabratus*) and *C. kefyr* (*Kluyveromyces marxianus*) belong to Family: Saccharomycetaceae. Family: Pichiaceae encompassing *C. krusei* (*Pichia kudriavzevii*). Family: Metschnikowisaceae including *C. lusitaniae* (*Clavispora lusitaniae*) [57]. *Trichosporon asahii* belongs to Division: Basidiomycota, Class: Tremellomycetes, Order: Trichosporonales, and Family: Trichosporonaceae [58].

The main causative agent of thrush was *C. albicans* as reported by Armstrong-James et al. [59]. Hussein et al. also stated that *C. albicans* represents the most prevalent member of the human microbiota within the oral cavity [60]. *C. albicans* is the most hazardous species in hospitalized patients and is responsible for invasive candidiasis with a mortality rate of about 50% [61, 62]. This agrees with our study, *C. albicans* is the most collected species and appeared in about 51.90% of the total isolates, only one isolate was detected of *C. guilliermondii* (*Meyerozyma guilliermondii*), *C. lusitaniae* (*Clavispora lusitaniae*), *C. parapsilosis*, *C. kefyr* (*Kluyveromyces marxianus*), and *Trichosporon asahii*. *K. marxianus* was isolated from the oral cavity of healthy individuals in a small proportion as described by Al-laaeiby et al. [63]. Although the general rate of *Trichosporon* infections is low, it is regarded as the second most common fungal infection [64].

There was a great resemblance between *C. albicans* and *C. dubliniensis* as mentioned by Sullivan et al. [65], in many phenotypic, morphological, and physiological characteristics. It was reported that at least 1.2-2% of *C*. *dubliniensis* were identified initially as *C. albicans* [66], and this aligns with our study.

From our investigation, we found that there was an overlap in the colors of some species when grown on Chromogenic and HiChrome agar media, and this agreed with Ozcan et al. [67], when mentioned that *C. albicans* sometimes failed to appear the expected color and might give an incorrect color and also found that definitive medium such Chromogenic agar was limited for identification of only three species of *Candida* (*albicans*, *krusei*, and *tropicalis*) with disputable value. Other morphological characterizations which help in the identification appeared under the microscope, *C. albicans* and *C. dubliniensis* can form true hyphae, *C. tropicalis* can form pseudo-hyphae under microscope, and the rest of species has no ability to form hyphae or pseudo-hyphae.

Molecular technique was used as an accurate means; specific primer (*CALB1-CALB2*) was selected for the identification of *C. albicans* as the most abundant species that repeated in the morphological identification, this primer was positive for *C. albicans* and negative for the other species as described by Luo and Mitchell [68]. *C. albicans* specific primer with germ tube test helped us to differentiate between *C. albicans* and *C. dubliniensis*. All isolates of *C. albicans* could form germ tube within two to three hours of incubation at 37 °C in 0.5 ml of human serum; under the same conditions, *C. krusei*, *C. parapsilosis*, and *C. tropicalis* did not have this ability [69]. *C. dubliniensis* is the only *non-albicans* species of *Candida* that can produce a germ tube [70]. The rest of the species were identified using (*CandF - CandR*) primers, and sequencing of some isolates with ITS region was also used for the precise identification.

Hydrolytic enzyme secretion is one of the most important virulence factors in *C. albicans* [71]. Ingham et al. [72] have shown that there is a correlation between the rise in secretions and the activities of hydrolytic enzymes, and the subsequent increase in the pathogenicity of *Candida* spp., resulting in the development of severe thrush. *C. albicans* isolates demonstrated a significant abundance of isolates that produce hemolysin, phospholipase, and proteinase. The most secreted enzyme by *Candida* spp. and other yeast-like structures in our study was proteinase, with 92.41%; it was tested using the method described by Humaid et al. [73] on filamentous fungi. The high level of proteinase secretion expressed by *C. parapsilosis*, followed by *P. kudriavzevii* although the *SAP1* gene was not detected in these isolates. It was reported that *C. parapsilosis*, as one of *C. non-albicans* that are isolated from surface infections, exhibits a broad spectrum of *SAPs* rather than those isolated from systemic infections [74].

The *SAP1* gene was positive in 41.8% of our isolates, most of them were *C. albicans* followed by a few species of *C. dubliniensis*, *N. glabratus*, and *C. tropicalis*. The adhesion of *C. tropicalis* to the target cell depends upon the activity of *SAP* genes [75]. All *SAP* genes are highly expressed by *C. albicans in vivo* during the oral infection, although there is different expression of specific hydrolytic enzymes [28]. Not all species express *SAP1*, although 92.41% of strains secreted proteinase enzyme, that’s means they may express other genes associated to proteinase production. *C. albicans* possesses a set of 10 *SAP* genes, characterized into distinct groups based on their sequence resemblances and functional pH preferences. These groups include *SAP1* to *SAP3*, *SAP4* to *SAP6*, *SAP7*, *SAP8*, and *SAP9* to *SAP10* [76, 77]. Studies indicate that *SAP1* to *SAP6*, and *SAP8* in *C. albicans* display a broad substrate specificity, qualifying them to degrade a wide range of host proteins, contributing to fungal virulence and adaptability. In contrast, *SAP7*, *SAP9*, and *SAP10* display more selective enzymatic activity [78–80]. Proteinases secreted by *Candida* play a significant role in the modulation of the host immune system response, influencing the activity of immune cells that are directly involved in defending against pathogens [81]. The pathogenic *Candida* spp. such as *C. dubliniensis*, *C. tropicalis*, and *C. parapsilosis* have been revealed *SAP* genes [82, 83]. Few pathogenic or nonpathogenic *Candida* spp. do not produce significant volumes of proteinase, although they may exhibit aspartyl proteinase genes [82].

The phospholipase enzyme was secreted by 70.9% of strains, and high levels of secretion appeared in *C. albicans*, which showed high expression of *PLB1* gene. Hemolysin was secreted with 69.6% of isolates in high levels in *N. glabratus*, *C. tropicalis*, and *K. marxianus*. Rossoni et al. [84] reported that *C. glabrata* exhibited the highest hemolytic activity, followed by *C. albicans*. *M. guilliermondii*, which didn’t secrete any of the enzymes despite the detection of the *PLB1* gene; this agreed with the study of Al-laaeiby et al. [63], who mentioned that *C. guilliermondii* displayed low phospholipase secretion and couldn’t produce proteinase. Also, it couldn’t produce hemolysin according to Rossoni et al. [84]. It is expected to be a weak strain in comparison with other species, and this agrees with Marcos-Zambrano et al. [85] in their study, who mentioned that *C. guilliermondii* is a low virulence strain as it was limited in biofilm formation in comparison with other strains of *Candida*. The *PLB1* gene was positive in 81% of strains and this proportion is close to that of strains isolated from the vagina of diabetic women which was reported by Bassyouni et al. [86]. The *PLB1* gene was detected in 87.5% of *Candida* isolates from women with diabetes and in 95% of isolates from women without diabetes. Naglik et al. [28] also stated that *SAP1* and *PLB1* are exhibited through active vaginal and oral thrush professionally. In this study, *C. albicans* displayed a strong expression of *PLB1* and *SAP1*, as it was the most dominant species observed. Another study agrees with our study, *C. albicans* demonstrates a high prevalence of *SAP1*, and *PLB1* genes according to Shrief et al. [87]. Other strains that don’t exhibit *PLB1*and secreted phospholipase may exhibit *PLB2* gene. Phospholipase enzymes, produced by the *PLB2* and *PLC1* genes, are thought to play more prominent roles in invasive infections and infections within the oral cavity, respectively [88].

Our investigation aims to detect the ability of isolated *Candida* spp. to produce 3 types of hydrolytic enzymes: protease, phospholipase, and hemolysin as indicators of their virulent efficacy, facilitating their attachment to the host cells and causing infection. There is no correlation between studying the virulence of *Candida* in host cells and in vitro, on epithelial cells, as the immune system may combat it by secretion antibodies, competition between *Candida* and other microorganisms in the host may occur, and these are completely absent on the epithelial cells in tissue culture. Also, the secretion of hydrolytic enzymes means that adhesion has already occurred. Previous research has supported our belief that when *Candida* adheres to host surfaces, it subsequently secretes hydrolytic enzymes. These enzymes facilitate the penetration and invasion of host tissues while also causing direct damage to the host cell membranes [89]. Several factors, such as interactions between various tissues or organs and competition with other microorganisms, can affect the colonization, survival, and commensal state of *Candida albicans*, ultimately impacting its virulence [90].

Secretion of hydrolytic enzymes is present in *Candida albicans*. They facilitate commensal and pathogenic characteristics, such as attachment to host tissue and causing the host cell membrane’s rupture. Because of these enzymes, invasion into the surfaces of mucous membranes and blood vessels is possible, and they also participate in avoiding the host’s immune response. The three main enzymes produced by *C. albicans* are *SAP* (secreted aspartyl protease), phospholipase, and hemolysin [91].

In addition to the virulence factors, knowledge of the essential predisposing factors for the development of candidiasis is also necessary, such as immunosuppression, neutropenia, age, diabetes, as well as information related to catheter use, patient care, long-term hospitalization, surgery, and long-term antimicrobial therapy [92].

*Candida albicans* interacts with epithelial cells at the mucosal membrane, playing a significant role in both commensalism and pathogenicity. It adheres and invades epithelial cells through induced endocytosis or active penetration, both of which rely on hypha formation. These processes can lead to cell damage via necrosis or apoptosis, though the exact mechanisms contributing to pathogenicity remain unclear. Additionally, fungal-epithelial interactions trigger innate immune responses, particularly through the recognition of hyphae via a MAPK-based signaling mechanism involving c-Fos transcription factor activation. This response, influenced by hyphal burden, functions as a ‘danger response’, warning the host of excessive fungal presence. Activated epithelial cells secrete proinflammatory cytokines and chemokines, recruiting immune cells like neutrophils, dendritic cells, and T cells. This immune response may lead to either clearance of the infection or a return to a non-activator colonization state. Clinically, targeting epithelial signaling pathways or fungal-specific mechanisms could pave the way for novel therapeutic strategies against mucosal fungal infections [93, 94].

Epithelial cells play a pivotal role in the initial stages of infection development. Their response influences subsequent reaction from the other cells, like immune cells. The signaling molecules released by host cells facilitate intercellular communication orchestrating the overall inflammatory response. Additionally, environmental factors and diseases can profoundly affect the organism’s homeostatic balance. The fungal commensal *Candida albicans* becomes pathogenic when host homeostasis is disrupted, which is associated with impairment of host immunity, causing mild superficial and serious systemic candidiasis [95].

According to previous studies, *Candida* species, including *C*. *albicans*, *C*. *glabrata*, *C*. *dubliniensis*, *C*. *tropicalis*, *C*. *parapsilosis*, and *C*. *krusei*, are commonly found in the oral flora of all humans [96–98]. While these fungi are typically harmless, they can sometimes lead to opportunistic infections when certain risk factors come into play. These factors may include poor oral hygiene, the use of mobile prosthetic devices, orthodontic appliances, and obturators, as well as conditions like dry mouth (xerostomia), smoking, and the use of steroid inhalers. Additionally, a carbohydrate-rich diet, certain oral mucosal diseases, pregnancy, and antibiotic or systemic corticosteroid therapies can contribute to susceptibility. Other relevant health concerns include tumors and their treatments, digestive system diseases, nutritional deficiencies (such as iron, folic acid, and vitamin deficiencies), various endocrinopathies (like diabetes and hypothyroidism), autoimmune disorders (such as Sjögren syndrome), HIV, and primary immunodeficiencies [99, 100]. Neonates with weakened immune systems, exposure to broad-spectrum antibiotics, an underdeveloped epithelial barrier, and frequent invasive procedures face a heightened risk of opportunistic fungal infections [101].

The main virulence factors of *Candida* spp. are the secretion of hydrolytic enzymes. The enzyme production was already higher in patients than in healthy individuals, to assist *Candida* in causing infection, a recent study by Maheronnagsh et al. [102] found that the activity levels of phospholipase, proteinase, and hemolysin in *Candida* species are notably elevated in patients undergoing chemotherapy compared to healthy people. Specifically, the hemolytic activity in these patients was significantly greater than that observed in healthy subjects (*P* < 0.05). Furthermore, diabetic patients exhibited higher enzyme production than their healthy counterparts [31].

In conclusion, *C. albicans*, *C. dubliniensis*, *P. kudriavzevii*, *C. tropicalis*, *N. glabratus*, *M. guilliermondii*, *C. lusitaniae*, *C. parapsilosis*, and *K. marxianus* belonged to Division: Ascomycota. *T. asahii*, which belongs to Division: Basidiomycota, was associated with thrush in neonates. *C. albicans* is the predominant cause of the infection in neonates compared to *non-albicans* species of *Candida*. The difference between two similar species of *C. albicans* and *C. dubliniensis* via the application of PCR techniques, such as specific primers (*CALB1*-*CALB2*), general primers (*CandF* - *CandR*), and sequencing techniques, was resolved for the first time in this study. Germ test tube was also the difference between (*C. albicans* and *C. dubliniensis*) and other yeasts associated with neonates’ thrush. *Trichosporon asahii* was observed for the first time as a causative agent of oral thrush in Egypt, to our knowledge, indicating that *Candida* is not the sole microorganism responsible for the condition. The other part of our investigation involves the possible treatment of neonatal thrush via *Boswellia carterii* and *Rosmarinus officinalis* aqueous extracts. *PLB1* and *SAP1* genes were associated with the secretion of phospholipase and proteinase enzymes, respectively. The isolates that didn’t exhibit any of *PLB1* or *SAP1* may exhibit other genes that promote phospholipase production, such as *PLB2*, or promote proteinase production, such as *SAP2*-*SAP10*. Other isolates with inactive *PLB1* or *SAP1* genes don’t produce phospholipase or proteinase enzymes. Furthermore, future studies must involve the detection of all hydrolytic enzymes biosynthesis genes.

## Data Availability

The datasets generated and/or analysed during the current study are deposited and available in the GenBank, https://www.ncbi.nlm.nih.gov/genbank/, with accession numbers: PP475375, PP475376, PP475377, PP475378, PP475379, PP475380, PP475381, PP475382, PP475383, PP475384, and PP475385.

## Abbreviations

PLB1: Phospholipase B1
SAP1: Secreted Aspartyl Proteinase 1
ITS: Internal Transcribed Spacer
CTAB: Cetyltrimethylammonium Bromide
Pz: Enzymatic Activity
PLB1: Phospholipase B1
SAP1: Secreted Aspartyl Proteinase
ICU: Intensive Care Unit
PCA: Principal Component Analysis
PSB: Potato Sucrose Broth

## References

[1] Slavkin HC. Streptococcus mutans, early childhood caries and new opportunities. J Amer Dent Ass. 1999;130:12: 1787–92. 10.14219/jada.archive.1999.0138. 10599184 10.14219/jada.archive.1999.0138

[2] Kadir T, Uygun B, Akyuz S. Prevalence of *Candida* species in Turkish children: relationship between dietary intake and carriage. Arch Oral Biol. 2005;50:33–7. 10.1016/j.archoralbio.2004.07.004. 15598415 10.1016/j.archoralbio.2004.07.004

[3] Hall RA, Noverr MC. Fungal interactions with the human host: exploring the spectrum of symbiosis. Curr Opin Microbiol. 2017;40:58–64. 10.1016/j.mib.2017.10.020. 29132066 10.1016/j.mib.2017.10.020 PMC5733695

[4] Qadir MI, Asif H. An overview to candidiasis-a Candida infection. Int J Advan Res Microbiol Immunol. 2020;2:1.

[5] Sarifakioglu E, Gunduz C, Gorpelioglu C. Oral mucosa manifestations in 100 pregnant versus non-pregnant patients: an epidemiological observational study. Eur J Dermatol. 2006;16(6):674–6. 10.1684/ejd.2006.0011. 17229610

[6] Lyu X, Zhao C, Yan ZM, Hua H. Efficacy of Nystatin for the treatment of oral candidiasis: a systematic review and meta-analysis. Drug Des Devel Ther. 2016;10:1161–71. 10.2147/DDDT. S100795. 27042008 10.2147/DDDT.S100795 PMC4801147

[7] Khadija B, Abbasi A, Khan S, Nadeem M, Badshah L, Faryal R. Isolation of pathogenic Candida species from oral cavity of postpartum females, and its association with obstetric and dental problems. Microb Pathog. 2019;131:40–6. 10.1016/j.micpath.2019.03.022. 30905714 10.1016/j.micpath.2019.03.022

[8] Dobias B. Moniliasis in pediatrics. Amer J Dis Child. 1957;94:3: 234–51. 10.1001/archpedi.1957.04030040016004. 10.1001/archpedi.1957.04030040016004 13457652

[9] Baley JE. Neonatal candidiasis: the current challenge. Clin Perinatol. 1991;18(2):263–80. 10.1016/S0095-5108(18)30523-. 1879108

[10] Riordan KJ, Wambach, editors. Breastfeeding and human lactation Jones & Bartlett Learning. J of Med 2010; 120:10: 871–879.

[11] Howell A, Isaacs D, Halliday R, Australasian Study Group For Neonatal Infections. Oral Nystatin prophylaxis and neonatal fungal infections. Arch Dis Childhood-Fetal Neonatal Ed. 2009;94(6):F429–33. 10.1136/adc.2008.157123. 19321509 10.1136/adc.2008.157123

[12] Ben Abdeljelil J, Saghrouni F, Khammari I, Gheith S, Fathallah A, Ben Said M, Boukadida J. Investigation of a cluster of *Candida albicans* invasive candidiasis in a neonatal intensive care unit by Pulsed- Field Gel Electrophoresis. The Sci World J. 2012; 2012:1: 138989. 10.1100/2012/138989 10.1100/2012/138989 PMC3322649 22547975

[13] Ben Abdeljelil J, Saghrouni F, Nouri S, Geith S, Khammari I, Fathallah A, Sboui H. & Ben saïd M. Neonatal invasive candidiasis in Tunisian hospital: incidence, risk factors, distribution of species and antifungal susceptibility. Mycoses 2012b 55:6: 493–500. 10.1111/j.1439-0507.2012.02189.x 10.1111/j.1439-0507.2012.02189.x 22448706

[14] Odds FC. Candida and candidosis: A review and bibliography. London: Bailliere Tindall; 1988.

[15] Terezhalmy GT, Huber MA. Oropharyngeal candidiasis: etiology, epidemiology, clinical manifestations, diagnosis, and treatment. Crest Oral-B at Dentalcare.com Contin. Educ Course 2011; p 1–16.

[16] Russell CM, Schaefer KG, Dixson A, Gray ALH, Pyron RJ, Alves DS, Moore N, Conley EA, Schuck RJ, White TA, Do TD, King GM, Barrera FN. The Candida albicans virulence factor Candidalysin polymerizes in solution to form membrane pores and damage epithelial cells. Elife. 2022;11:e75490. 10.7554/eLife.75490. 36173096 10.7554/eLife.75490 PMC9522247

[17] Richardson JP, Brown R, Kichik N, Lee S, Priest E, Mogavero S, Maufrais C, Wickramasinghe DN, Tsavou A, Kotowicz NK, Hepworth OW, Gallego-Cortés A, Ponde NO, Ho J, Moyes DL, Wilson D, D’Enfert C, Hube B, Naglik JR. Candidalysins are a new family of cytolytic fungal peptide toxins. mBio. 2022;13(1):e03510–21. 10.1128/mbio.03510-21. 35073742 10.1128/mbio.03510-21 PMC8787473

[18] Silva RB, Fusco-Almeida AM, Matsumoto MT, Baeza LC, Benaducci T, Mendes-Giannini MJS. Genetic diversity and antifungal susceptibility testing of trichosporon Asahii isolated of intensive care units patients. Braz J Microbiol. 2008;39(3):585–92. 10.1590/S1517-83822008000300033. 24031270 10.1590/S1517-838220080003000033 PMC3768427

[19] Silva S, Negri M, Henriques M, Oliveira R, Williams DW, Azeredo J. Candida glabrata, Candida parapsilosis and Candida tropicalis: biology, epidemiology, pathogenicity and antifungal resistance. FEMS Microbiol Rev. 2011;36(2):288–305. 10.1111/j.1574-6976.2011.00278.x. 21569057 10.1111/j.1574-6976.2011.00278.x

[20] Jørgensen MR. Pathophysiological microenvironments in oral candidiasis. APMIS. 2024. 10.1111/apm.13412. 38571459 10.1111/apm.13412

[21] Taschdjian CL, Burchall JJ, Kozinn PJ. Rapid identification of Candida albicans by filamentation on serum and serum substitutes. AMA J Dis Child. 1960;99:212–5. 10.1001/archpedi.1960.02070030214011. 13836995 10.1001/archpedi.1960.02070030214011

[22] Desai JV. Candida albicans hyphae: from growth initiation to invasion. J Fungi (Basel). 2018;4:10. 10.3390/jof4010010. 29371503 10.3390/jof4010010 PMC5872313

[23] Gutierrez J, Morales P, Gonzalez MA, Quindos G. Candida Dubliniensis, a new fungal pathogen. J. of basic microbiol: an int. J. on biochem, physiol, gene, morph, 182 and ecology of microorganisms. 2002; 42:3: 207–27. 10.1002/1521-4028 10.1002/1521-4028(200206)42:3<207::AID-JOBM207>3.0.CO;2-C 12111748

[24] Almeida RS, Wilson D, Hube B. Candida albicans iron acquisition within the host. FEMS Yeast Res. 2009;9:7:1000–12. 10.1111/j.1567-1364.2009.00570.x. 19788558 10.1111/j.1567-1364.2009.00570.x

[25] Almeida RS, Brunke S, Albrecht A, Thewes S, Laue M, Edwards JE, Filler SG, Hube B. The hyphal-associated adhesin and Invasin Als3 of Candida albicans mediates iron acquisition from host ferritin. PLoS Pathog. 2008;4:11. 10.1371/journal.ppat.1000217. 10.1371/journal.ppat.1000217 PMC2581891 19023418

[26] Barrett-Bee K, Hayes Y, Wilson RG, et al. A comparison of phospholipase activity, cellular adherence and pathogenicity of yeasts. J Gen Microbiol. 1985;131:1217–21. 10.1099/00221287-131-5-1217. 3894572 10.1099/00221287-131-5-1217

[27] Mukherjee PK, Ghannoum MA. Secretory proteins in fungal virulence. In: Calderone RA, Cihlar RL, editors. Fungal pathogenesis: principles and clinical applications. New York: Marcel Dekker; 2002. pp. 51–79.

[28] Naglik JR, Rodgers CA, Shirlaw PJ, Dobbie JL, Fernandes-Naglik LL, Greenspan D, Agabian N, Challacombe SJ. Differential expression of *Candida albicans* secreted aspartyl proteinase and phospholipase B genes in humans correlates with active oral and vaginal infections. J Infect Dis. 2003;188(3):469–79. 10.1086/376536. 12870130 10.1086/376536

[29] Samaranayake YH, Dassanayake RS, Cheung BPK, Jayatilake JAMS, Yeung KWS, Yau JYY, Samaranayake LP. Differential phospholipase gene expression by Candida albicans in artificial media and cultured human oral epithelium. APMIS. 2006;114(12):857–66. 10.1111/j.1600-0463.2006.apm_479.x. 17207086 10.1111/j.1600-0463.2006.apm_479.x

[30] Mahmoudabadi AZ, Zarrin M, Miry S. Phospholipase activity of Candida albicans isolated from vagina and urine samples. Jundishapur J Microbiol. 2010;3:169–73.

[31] Tsang CSP, Chu FCS, Leung WK, Jin LJ, Samaranayake LP, Siu SC. Phospholipase, proteinase and haemolytic activities of Candida albicans isolated from oral cavities of patients with type 2 diabetes mellitus. J Med Microbiol. 2007;56(10):1393–8. 10.1099/jmm.0.47303-0. 17893179 10.1099/jmm.0.47303-0

[32] Monika S, Małgorzata B, Zbigniew O. Contribution of aspartic proteases in Candida virulence. Protease inhibitors against *Candida* infections. Curr Protein Pept Sci. 2017;18:10:1050–62. 10.2174/1389203717666160809155749. 27514853 10.2174/1389203717666160809155749

[33] Hube B, Ruchel R, Monod M, Sanglard D, Odds FC. Functional aspects of secreted Candida proteinases. Adv Exp Med Biol. 1998;436:339–44. 9561239 10.1007/978-1-4615-5373-1_47

[34] Carlier GIM. An All-British mycological culture media. Brit J Derm Syph. 1948;60:2. 10.1111/j.1365-2133.1948.tb10992.x 18904997

[35] Sabouraud R. Contribution a L’etude de La trichophytie Humaine. Etude clinique, microscopique et bacterioloqique Sur La pluralité des trichophytons de L’homme. Annals Dermatology Syphilis. 1892;3:1061–87.

[36] Gams W, Hoekstra ES, Aptroot ACBS. Course on Mycology. Centraalbureau voor Schimmelcultures, AG Baarn, the Netherlands; 1998.

[37] Odds FC, Bernaerts R. CHROMagar Candida, a new differential isolation medium for presumptive identification of clinically important yeast species. J Clin Microbiol. 1994;32:1923–9. 10.1128/jcm.32.8.1923-1929.1994. 7989544 10.1128/jcm.32.8.1923-1929.1994 PMC263904

[38] Freydiére AM. Evaluation of CHROMagar Candida plates. J Clin Microbiol. 1996;34:2048. 8818913 10.1128/jcm.34.8.2048-2048.1996 PMC229185

[39] Perry JL, Miller GR. Umbelliferyl-labeled galactosaminide as an aid in identification of Candida albicans. J Clin Microbiol. 1987;25:12: 2424–5. 10.1128/jcm.25.12.2424-2425.1987. 3323233 10.1128/jcm.25.12.2424-2425.1987 PMC269508

[40] Rousselle P, Freydiere AM, Couillerot PJ, De Montclos H, Gille Y. Rapid identification of *Candida albicans* by using Albicans ID and fluoroplate agar plates. J Clin Microbiol. 1994;32(12):3034–6. 10.1128/jcm.32.12.3034-3036.1994. 7883894 10.1128/jcm.32.12.3034-3036.1994 PMC264220

[41] Makwana DGE, Gadhavi DH, Sinha DM. Comparison of germ tube production by Candida albicans in various media: comparison of germ tube production by Candida albicans. NJIRM. 2012;3:2:6–8.

[42] Doyle JJ, Doyle JL. A rapid DNA isolation procedure for small quantities of fresh leaf tissue. Phytochem Bull. 1987;19:11–5.

[43] White TJ, Bruns T, Lee SJWT, Taylor J. Amplification and direct sequencing of fungal ribosomal RNA genes for phylogenetics. PCR protocols: a guide to methods and applications. 1990; 18:1: 315–322.

[44] García-Salazar E, Acosta-Altamirano G, Betancourt-Cisneros P, Reyes-Montes MDR, Rosas-De-Paz E, Duarte-Escalante E, Sánchez-Conejo AR. Ocharan Hernández E, Frías-De-León MG. Detection and molecular identification of eight Candida species in clinical samples by simplex PCR. Microorganisms. 2022;10(2):374. 10.3390/microorganisms10020374. 35208828 10.3390/microorganisms10020374 PMC8880469

[45] Zhang L, Zhao C, Zhu D, Ohta Y, Wang Y. Purification and characterization of Inulinase from *Aspergillus Niger* AF10 expressed in Pichia pastoris. Protein Expr Purif. 2004;35(2):272–5. 10.1016/j.pep.2004.02.015. 15135402 10.1016/j.pep.2004.02.015

[46] Moeller M, Maurer H, Roesler M. MDMA in blood, urine and hair: a forensic case. In Proceedings, 30th TIAAFT Meeting. Fukouka, Japan: Yoyodo Printing Kaisha Ltd. 1992; 56–61.

[47] Malcok HK, Aktas E, Ayyildiz A, Yigit N, Yazgi H. Hemolytic activities of the Candida species in liquidmedium. Eur J Med. 2009;41(2):95–8. PMC4299835 25610076

[48] Price MF, Wilkinson ID, Gentry LO. Plate method for detection of phospholipase activity in Candida albicans. Sabouraudia: J Med Veterinary Mycol. 1982;20(1):7–14. 10.1080/00362178285380031. 10.1080/00362178285380031 7038928

[49] Paterson RRM, Bridge PD. Biochemical techniques for filamentous fungi (IMI techanical Handbook). Oxford University Press; 1994.

[50] Ozturkoglu-Budak S, Wiebenga A, Bron PA, de Vries RP. Protease and lipase activities of fungal and bacterial strains derived from an artisanal Raw Ewe’s milk cheese. Int J Food Microbiol. 2016;237:17–27. 10.1016/j.ijfoodmicro.2016.08.007. 27541978 10.1016/j.ijfoodmicro.2016.08.007

[51] Kumar M. Thrush. Background, pathophysiology, epidemiology. Available from: http://emedicine.medscape.com/article/969147-overview. Published January 6, 2017. Accessed September 26, 2016.

[52] Dermitzaki N, Baltogianni M, Tsekoura E, Giapros V. Invasive Candida infections in neonatal intensive care units: risk factors and new insights in prevention. Pathogens. 2024;6(8):13. 10.3390/pathogens13080660. 10.3390/pathogens13080660 PMC11356907 39204260

[53] Battersby AJ, Khara J, Wright VJ, Levy O, Kampmann B. Antimicrobial proteins and peptides in early life: ontogeny and translational opportunities. Front Immunol. 2016;18:7309. 10.3389/fimmu.2016.00309. 10.3389/fimmu.2016.00309 PMC4989132 27588020

[54] Benjamin DK Jr, Stoll BJ, Gantz MG, Walsh MC, Sánchez PJ, Das A, Shankaran S, Higgins RD, Auten KJ, Miller NA, Walsh TJ. Neonatal candidiasis: epidemiology, risk factors, and clinical judgment. Pediatrics. 2010;1(1264):e865–73. 10.1542/peds.2009-3412. 10.1542/peds.2009-3412 PMC3045840 20876174

[55] Sharma M, Chakrabarti A. Candidiasis and other emerging yeasts. Cur Fung Infect Rep. 2023;17:1:15–24. 10.1007/s12281-023-00455-3. 10.1007/s12281-023-00455-3 PMC9886541 36741271

[56] Berkhout. De schimmelgesl. Monilia, oidium, oospora En Torula. Disset Ultrecht. 1923; 44.

[57] Takashima M, Sugita T. Taxonomy of pathogenic yeasts *Candida*, *Cryptococcus*, *Malassezia*, and *Trichosporon* current status, future perspectives, and proposal for transfer of six Candida species to the genus Nakaseomyces. Med Mycol J. 2022;63:4:119–32. 10.3314/mmj.22.004. 36450564 10.3314/mmj.22.004

[58] Behrend G. Ueber trichomycosis nodosa (Juhel-Rénoy): Piedra (Osorio). Berliner Klinische Wochenschrift. 1890; 27: 464–7.

[59] Armstrong-James D, Brown GD, Netea MG, Zelante T, Gresnigt M, van de Veerdonk F, et al. Immunotherapeutic approaches to treatment of fungal diseases. Lancet Infect Dis. 2017;17:e393–402. 10.1016/S1473-3099(17)30442-5. 28774700 10.1016/S1473-3099(17)30442-5

[60] Hussein NA, Abdel-Sater MA, Edrees MF, Nasr AM, Danial AW. Mycobiota associated with teeth and oral cavity and their relevant to dental and periodontal diseases. Assiut Univ J Multidisciplinary Sci Res. 2023;52(2):195–231. 10.21608/aunj.2023.202022.1047.

[61] Richardson JP, Brown R, Kichik N, Lee S, Priest E, Mogavero S, Maufrais C, Wickramasinghe DN, Tsavou A, Kotowicz NK, et al. Candidalysins are a new family of cytolytic fungal peptide toxins. MBio. 2022;13(1):e03510–21. 10.1128/mbio.03510-21. 35073742 10.1128/mbio.03510-21 PMC8787473

[62] Russell CM, Schaefer KG, Dixson A, Gray AL, Pyron RJ, Alves DS, Moore N, Conley EA, Schuck RJ, White TA, Do TD. The Candida albicans virulence factor Candidalysin polymerizes in solution to form membrane pores and damage epithelial cells. Elife. 2022;2911:e75490. 10.7554/eLife.75490. 10.7554/eLife.75490 PMC9522247 36173096

[63] Al-laaeiby AIE, Al-Mousawi AA, Alrubayae I, Al-Saadoon A, Almayahi M. Innate pathogenic traits in oral yeasts. Karbala Int J Mod Sci. 2020;6(4):375–84. 10.33640/2405-609X.1984.

[64] Girmenia C, Pagano L, Martino B, D’Antonio D, Fanci R, Specchia G, Melillo L, Buelli M, Pizzarelli G, Venditti M, Martino P. Invasive infections caused by trichosporon species and geotrichum capitatum in patients with hematological malignancies: a retrospective multicenter study from Italy and review of the literature. J Clin Microb. 2005;43(4):1818–28. 10.1128/jcm.43.4.1818-1828.2005. 10.1128/JCM.43.4.1818-1828.2005 PMC1081342 15815003

[65] Sullivan DJ, Moran GP, Pinjon E, Al-Mosaid A, Stokes C, Vaughan C, Coleman DC. Comparison of the epidemiology, drug resistance mechanisms, and virulence of Candida Dubliniensis and Candida albicans. FEMS Yeast Res. 2004;4:4–5. 10.1016/S1567-. 1356(03)00240-X. 10.1016/S1567-1356(03)00240-X 14734017

[66] Jabra-Rizk MA, Falkler WA Jr, Merz WG, Baqui AA, Kelley JI, Meiller TF. Retrospective identification and characterization of *Candida Dubliniensis* isolates among *Candida albicans* clinical laboratory isolates from human immunodeficiency virus (HIV)- infected and non-HIV-infected individuals. J Clin Microbiol. 2000;38:2423–6. 10.1128/jcm.38.6.2423-2426.2000. 10835022 10.1128/jcm.38.6.2423-2426.2000 PMC86831

[67] Ozcan K, Ilkit M, Ates A, Turac-Bicer A, Demirhindi H. Performance of chromogenic Candida agar and CHROMagar Candida in recovery and presumptive identification of monofungal and polyfungal vaginal isolates. Med Myco. 2010;48(1):29–34. 10.3109/13693780802713224. 10.3109/13693780802713224 19191167

[68] Luo G, Mitchell TG. Rapid identification of pathogenic fungi directly from cultures by using multiplex PCR. J Clin Microbiol. 2002;40(8):2860–5. 10.1128/jcm.40.8.2860-2865.2002. 12149343 10.1128/JCM.40.8.2860-2865.2002 PMC120665

[69] Hussein HS. Isolating and diagnosing some types of *Candida* spp yeast and studying their sensitivity to some antifungals. South Asian Res J Pharma Sci. 2024;6(4):124–30. 10.36346/sarjps.2024.v06i04.002.

[70] Sullivan D, Coleman D. Candida Dubliniensis: characteristics and identification. J Clin Microbiol. 1998;36(2):329–34. 10.1128/jcm.36.2.329-334.1998. 9466736 10.1128/jcm.36.2.329-334.1998 PMC104537

[71] Islam A. Characterizing genes of Candida albicans involved in invasiveness, mating and genotoxic stress response (Doctoral dissertation, Concordia University); 2018.

[72] Ingham CJ, Boonstra S, Levels S, de Lange M, Meis JF, Schneeberger PM. Rapid susceptibility testing and microcolony analysis of Candida spp. Cultured and imaged on porous aluminum oxide. PLoS ONE. 2012;7:3: e33818. 10.1371/journal.pone.0033818. 22439000 10.1371/journal.pone.0033818 PMC3306290

[73] Humaid ARA, Al-Ghalibi SMS, Abdel-Sater MA, Salahaddin RH. Proteases and lipases activity of fungi isolated from local cheese, Republic of Yemen. Ass Univ Bull Environ Res. 2020; 23.

[74] Alwaily ER, Flaih MH, Abood MS, Hussein KR. Determination of fungal and parasitic infections caused vaginitis: molecular identification of *Candida* parapsilosis in Al-Nasiriyah City, Iraq. Bagh Sci J. 2023;20(3):0778–0778. 10.21123/bsj.2022.6744.

[75] Kontoyiannis DP, Vaziri I, Hanna HA, Boktour M, Thornby J, Hachem R, Bodey GP, Raad II. Risk factors for Candida tropicalis fungemia in patients with cancer. Clin Infect Dis. 2001;15(10):33. 10.1086/323812. 10.1086/323812 11568858

[76] Hube B, Naglik J. Candida albicans proteinases: resolving the mystery of a gene family. Microbiology. 2001;147(8):1997–2005. 10.1099/00221287-147-8-1997. 11495978 10.1099/00221287-147-8-1997

[77] Aoki W, Kitahara N, Miura N, Morisaka H, Yamamoto Y, Kuroda K, Ueda M. Comprehensive characterization of secreted aspartic proteases encoded by a virulence gene family in Candida albicans. J Biochem. 2011;1(4):431–8. 10.1093/jb/mvr073. 10.1093/jb/mvr073 21646240

[78] Koelsch G, Tang J, Loy JA, Monod M, Jackson K, Foundling SI, Lin X. Enzymic characteristics of secreted aspartic proteases of Candida albicans. Biochimica et biophysica acta (BBA)-Protein structure and molecul enzymo. 2000; 14;1480(1–2):117–31. 10.1016/S0167-4838(00)00068-6 10.1016/s0167-4838(00)00068-6 11004559

[79] Albrecht A, Felk A, Pichova I, Naglik JR, Schaller M, de Groot P, MacCallum D, Odds FC, Schäfer W, Klis F, Monod M. Glycosylphosphatidylinositol-anchored proteases of Candida albicans target proteins necessary for both cellular processes and host-pathogen interactions. J Biol Chem. 2006;13(2):281. 10.1074/jbc.M509297200. 10.1074/jbc.M509297200 16269404

[80] Schild L, Heyken A, de Groot PW, Hiller E, Mock M, de Koster C, Horn U, Rupp S, Hube B. Proteolytic cleavage of covalently linked cell wall proteins by Candida albicans Sap9 and SAP10. Euk Cell. 2011;10(1):98–109. 10.1128/ec.00210-10. 10.1128/EC.00210-10 PMC3019796 21097664

[81] Bras G, Satala D, Juszczak M, Kulig K, Wronowska E, Bednarek A, Zawrotniak M, Rapala-Kozik M, Karkowska-Kuleta J. Secreted aspartic proteinases: key factors in Candida infections and host-pathogen interactions. Int J Mole Sci. 2024;27(9):4775. 10.3390/ijms25094775. 10.3390/ijms25094775 PMC11084781 38731993

[82] Monod M, Togni G, Hube B, Sangland D. Multiplicity of genes encoding secreted aspartic proteinases in Candida species. Mol Microbiol. 1994;13:357–68. 10.1111/j.1365-2958.1994.tb00429.x. 7984113 10.1111/j.1365-2958.1994.tb00429.x

[83] Gilfillan D, Derek G, Parkinson JS, Coleman T, Gow DC. Candida Dubliniensis: phylogeny and putative virulence factors. Microb. 1998;144(4):829–38. 10.1099/00221287-144-4-829. 10.1099/00221287-144-4-829 9579058

[84] Rossoni RD, Barbosa JO, Vilela SFG, Jorge AOC, Junqueira JC. Comparison of the hemolytic activity between C. albicans and nonalbicans Candida species. Braz Oral Res. 2013;27(6):484–9. 10.1590/S1806-83242013000600007. 10.1590/S1806-83242013000600007 24346046

[85] Marcos-Zambrano LJ, Puig-Asensio M, Pérez-García F, Escribano P, Sánchez-Carrillo C, Zaragoza O, Padilla B, Cuenca-Estrella M, Almirante B, Martín-Gómez MT, Muñoz P, Bouza E, Guinea J. Candida guilliermondii complex is characterized by high antifungal resistance but low mortality in 22 cases of candidemia. Antimicrob Agents Chemother. 2017;61(7):10–1128. 10.1128/aac.00099-17. 10.1128/AAC.00099-17 PMC5487632 28438935

[86] Bassyouni RH, Wegdan AA, Abdelmoneim A, Said W, AboElnaga F. Phospholipase and aspartyl proteinase activities of Candida species causing vulvovaginal candidiasis in patients with type 2 diabetes mellitus. J Microbiol Biotech. 2015;25:10: 1734–41. 10.4014/jmb.1504.04009. 10.4014/jmb.1504.04009 26032358

[87] Shrief R, Zaki ME, El-Sehsah EM, Ghaleb S, Mofreh M. Study of antifungal susceptibility, virulence genes and biofilm formation in Candida albicans. The Open Microb J. 2019; 30;13(1). 10.2174/1874285801913010241

[88] Tunç B, Demiray Gürbüz EB, Doluca Dereli M. Investigation of phospholipase activity in Candida albicans strains isolated from blood and oral cavity specimens. Türk Mikrobiyoloji Cemiyeti Dergisi. 2021;51(1). 10.5222/tmcd.2021.84429.

[89] Lopes JP, Lionakis MS. Pathogenesis and virulence of Candida albicans. Virulence. 2022 31;13(1):89–121. 10.1080/21505594.2021.2019950 10.1080/21505594.2021.2019950 PMC9728475 34964702

[90] Navarro-García F, Sánchez M, Nombela C, Pla J. Virulence genes in the pathogenic yeast Candida albicans. FEMS microb rev. 2001; 1;25(2):245–68. 10.1111/j.1574-6976.2001.tb00577.x 10.1111/j.1574-6976.2001.tb00577.x 11250036

[91] Wibawa T. The role of virulence factors in Candida albicans pathogenicity. J Med Sci. 2016;48:58–68. 10.19106/JMedSci004801201606.

[92] Talapko J, Juzbašić M, Matijević T, Pustijanac E, Bekić S, Kotris I, Škrlec I. Candida albicans—the virulence factors and clinical manifestations of infection. J Fun. 2021;22(72):79. 10.3390/jof7020079. 10.3390/jof7020079 PMC7912069 33499276

[93] Naglik JR, Moyes DL, Wächtler B, Hube B. Candida albicans interactions with epithelial cells and mucosal immunity. Microb Infect. 2011;13(12–13):963–76. 10.1016/j.micinf.2011.06.009. 10.1016/j.micinf.2011.06.009 PMC3185145 21801848

[94] Ho J, Yang X, Nikou SA, Kichik N, Donkin A, Ponde NO, Richardson JP, Gratacap RL, Archambault LS, Zwirner CP, Murciano C. Candidalysin activates innate epithelial immune responses via epidermal growth factor receptor. Nat Communic. 2019;24(1):2297. 10.1038/s41467-019-09915-2. 10.1038/s41467-019-09915-2 PMC6534540 31127085

[95] Kulig K, Wronowska E, Juszczak M, Zawrotniak M, Karkowska-Kuleta J, Rapala-Kozik M. Host cell responses to Candida albicans biofilm-derived extracellular vesicles. Front Cell Infect Microb. 2025;14:1499461. 10.3389/fcimb.2024.1499461. 10.3389/fcimb.2024.1499461 PMC11772320 39877654

[96] Lewis MAO, Williams DW. Diagnosis and management of oral candidosis. Br Dent J. 2017;223:675–81. 10.1038/sj.bdj.2017.886. 29123282 10.1038/sj.bdj.2017.886

[97] Peters BA, Wu J, Hayes RB, Ahn J. The oral fungal mycobiome: characteristics and relation to periodontitis in a pilot study. BMC Microbiol. 2017;17:157. 10.1186/s12866-017-1064-9. 28701186 10.1186/s12866-017-1064-9 PMC5508751

[98] Aslani N, Janbabaei G, Abastabar M, Meis JF, Babaeian M, Khodavaisy S, Boekhout T, Badali H. Identification of uncommon oral yeasts from cancer patients by MALDI-TOF mass spectrometry. BMC Infect Dis. 2018;18:24. 10.1186/s12879-017-2916-5. 29310582 10.1186/s12879-017-2916-5 PMC5759378

[99] Baumgardner DJ. Oral fungal microbiota: to thrush and beyond. J. Patient-Cent. Res Rev. 2019;6:252–61. 10.17294/2330-0698.1705. 10.17294/2330-0698.1705 PMC6827844 31768404

[100] Serrano J, López-Pintor RM, Ramírez L, Fernández-Castro M, Sanz M, Melchor S, Peiteado D, Hernández G. Risk factors related to oral candidiasis in patients with primary Sjögren’s syndrome. Med Oral Patol Oral Cir Bucal. 2020;25:e700–5. 10.4317/medoral.23719. 32683379 10.4317/medoral.23719 PMC7473438

[101] Sokou R, Palioura AE, Kopanou Taliaka P, Konstantinidi A, Tsantes AG, Piovani D, Tsante KA, Gounari EA, Iliodromiti Z, Boutsikou T, et al. *Candida auris* infection, a rapidly emerging threat in the neonatal intensive care units: A systematic review. J Clinicl Med. 2024;13(6):1586. 10.3390/jcm13061586. 10.3390/jcm13061586 PMC10971333 38541815

[102] Maheronnagsh M, Fatahinia M, Dehghan P, Mahmoudabadi AZ, Kheirkhah M. Comparison of virulence factors of different Candida species isolated from the oral cavity of cancer patients and normal individuals. Jundishapur J Microbiol. 2019;12(5):e91556. 10.5812/jjm.91556.

